# Water Deficit History Selects Plant Beneficial Soil Bacteria Differently Under Conventional and Organic Farming

**DOI:** 10.3389/fmicb.2022.824437

**Published:** 2022-06-13

**Authors:** Lucie Gebauer, Claudia Breitkreuz, Anna Heintz-Buschart, Thomas Reitz, François Buscot, Mika Tarkka, Marie-Lara Bouffaud

**Affiliations:** ^1^Helmholtz Centre for Environmental Research, Halle, Germany; ^2^German Centre for Integrative Biodiversity Research (iDiv) Halle-Jena-Leipzig, Leipzig, Germany; ^3^Biosystems Data Analysis Group, Swammerdam Institute for Life Sciences, University of Amsterdam, Amsterdam, Netherlands

**Keywords:** drought legacy, ACC deaminase, PGPR, organic and conventional farming, wheat, barley, *acdS* gene, amplicon sequencing

## Abstract

Water deficit tolerance is critical for plant fitness and survival, especially when successive drought events happen. Specific soil microorganisms are however able to improve plant tolerance to stresses, such as those displaying a 1-aminocyclopropane-1-carboxylate (ACC) deaminase activity. Microorganisms adapted to dry conditions can be selected by plants over time because of properties such as sporulation, substrate preference, or cell-wall thickness. However, the complexity and interconnection between abiotic factors, like drought or soil management, and biotic factors, like plant species identity, make it difficult to elucidate the general selection processes of such microorganisms. Using a pot experiment in which wheat and barley were grown on conventional and organic farming soils, we determined the effect of water deficit history on soil microorganisms by comparing single and successive events of water limitation. The analysis showed that water deficit strongly impacts the composition of both the total microbial community (16S rRNA genes) and one of ACC deaminase-positive (*acdS*^+^) microorganisms in the rhizosphere. In contrast, successive dry conditions moderately influence the abundance and diversity of both communities compared to a single dry event. We revealed interactive effects of the farming soil type and the water deficit conditioning treatment. Indeed, possibly due to better nutrient status, plants grown on soils from conventional farming showed higher growth and were able to select more adapted microbial taxa. Some of them are already known for their plant-beneficial properties like the Actinobacteria *Streptomyces*, but interestingly, some Proteobacteria were also enriched after a water deficit history under conventional farming. Our approach allowed us to identify key microbial taxa promoting drought adaptation of cereals, thus improving our understanding of drought effects on plant-microbe interactions.

## Introduction

Plants interact with a large diversity of microorganisms in soils, especially in the rhizosphere, the zone directly influenced by the roots. The microbial communities differ between plant species or genotypes and may have various effects on plant health (Raaijmakers et al., 2009; Berendsen et al., 2012). To deal with different stressors, plants recruit beneficial microbes into their rhizospheres (Naylor and Coleman-Derr, 2018), which are summarized as plant growth-promoting rhizobacteria (PGPR). The action spectrum of these PGPR comprises a range of functional traits including biofertilization, root growth stimulation, pathogen suppression, rhizoremediation, and induction of systemic resistance. These processes often are the result of modifications in plant hormone production levels (Lugtenberg and Kamilova, 2009; Vacheron et al., 2013; Backer et al., 2018). Ethylene is a phytohormone that plays a central role in plant development and plant responses to stress conditions, especially at the root level (Tanimoto et al., 1995; Mattoo and Suttle, 2017). Since ethylene biosynthesis increases in response to environmental biotic and abiotic stresses, the production of ethylene can serve as an indicator of the susceptibility of plants to different stressors (Morgan and Drew, 1997; Balota et al., 2004). Lowering ethylene concentrations in stressed plants, the enzyme 1-aminocyclopropane-1-carboxylate (ACC) deaminase encoded by the *acdS* gene in some PGPR, degrades the ethylene precursor ACC to ammonia and α-ketobutyrate (Glick, 2005, 2014). This microbial ACC deaminase-based reduction of plant stress has already been demonstrated for different environmental stressors, such as flooding, drought, heat, cold, pathogen colonization, as well as high concentrations of salt, heavy metals, and organic pollutants (Gamalero and Glick, 2012).

In relation to the present global change, drought events are predicted to increase in frequency and intensity (Spinoni et al., 2018; Hari et al., 2020), which will have significant impacts on plant production as well as on biogeochemically relevant soil processes (Fahad et al., 2017; Canarini et al., 2021). Plants produce increased amounts of ethylene upon imposition of drought stress, and thereby, stress susceptible plants produce higher levels of ethylene than stress-tolerant ones (Balota et al., 2004). The stress releasing action of ACC deaminase containing (*acdS*^+^) PGPR strains is based on a negative influence on ethylene production on plant growth combined with the support of the antioxidative systems of the plant (Jaemsaeng et al., 2018; Gowtham et al., 2020; Murali et al., 2021). At the functional level, extensive literature exists on the positive effect of the inoculation of *acdS*^+^ PGPR strains to increase drought tolerance and mitigate drought stress in plants (e.g., Arshad et al., 2008; Shakir et al., 2012; Danish et al., 2020). However, these studies have been performed with a limited set of bacteria, whereas in nature plants are constantly interacting with a multitude of microorganisms with different functional properties. Plant and microbe partners involved in these interactions form the holobiont, which is considered as being the unit of selection in evolution (Zilber-Rosenberg and Rosenberg, 2008) driving acclimation and/or adaptation processes, especially under stress pressure.

Under drought conditions, plants exhibit an altered carbon allocation and root exudate composition (Sanaullah et al., 2012; Gargallo-Garriga et al., 2018), which results in the restructuring of rhizosphere microbial communities (Berg and Smalla, 2009; Santos-Medellín et al., 2017; Zhalnina et al., 2018; Canarini et al., 2021). Members of these restructured plant-associated microbiota can contribute to plant survival by fostering short-term acclimation through the production of phytohormones or exopolysaccharides (direct response) and long-term adaptation (after several plant growth cycles) to drought stress through the selection of an adapted microbial community (Lau and Lennon, 2012; Marasco et al., 2012; Vurukonda et al., 2016). Drought history, i.e., the consequences of recurrent drought events, may affect soil processes or plant performance *via* the impact on the soil microbial community (Canarini et al., 2021; Munoz-Ucros et al., 2022). The impact of drought history has been analyzed in terms of resistance (the ability of the community to tolerate the disturbance) and resilience (the ability of the community to recover from the disturbance after rewetting; Griffiths and Philippot, 2013) after short periods of drought (de Nijs et al., 2019; Veach and Zeglin, 2020; Leizeaga et al., 2021). The processes of resistance and resilience concern rapid responses during or directly following the stress. In contrast, long-term adaptation to stress implies the selection of adapted microbial communities harboring beneficial functions after recurrent stress events in order to better tolerate subsequent stress (Evans et al., 2014; Bastida et al., 2017). In this context, the role of the selection of adapted PGPR to support the plant under stress is unclear.

Plant–soil feedback describes the relative growth of a plant in its own conspecific soil, compared to its growth with heterospecific soil that has been conditioned by another plant species (Bever et al., 1997). Plant–soil feedback (PSF) occurs when plants alter soil properties such as nutrient availability or secondary metabolite spectra, but also modify plant-associated microbial communities (Bennett and Klironomos, 2019). PSF has traditionally been studied in greenhouse experiments and without considering abiotic or biotic drivers, although it has lately become evident that changes in the environment can affect both the strength and the direction of the PSF (De Long et al., 2019). For instance, the previous drought can not only modify PSF, affecting con- and heterospecific plant growth responses and mediating drought legacy effects on microbial communities, but also influence plant and microbial responses to subsequent drought (Kaisermann et al., 2017). By influencing the abundance and composition of plant beneficial microorganisms, for instance, that of the *acdS*^+^ population, plant-soil feedback and drought history could influence plant growth. Thus, a direct link between water deficit history effect on *acdS*^+^ plant-beneficial microbial communities and increased plant drought resistance remains to be substantiated (de Vries et al., 2020).

Recently an approach for quantification and characterization of these PGPR was developed by Bouffaud et al. (2018), which uses specific PCR primers to target ACC deaminase-producing microorganisms. The *acdS* gene is highly conserved among microorganisms (bacteria and micro-eukaryotes) and is thus a suitable marker to assess the ACC deaminase functional communities and dynamics in the context of drought. Studies based on this approach have shown that the abundance and diversity of *acdS*^+^ microorganisms were modulated by plant species and plant genotype (Bouffaud et al., 2018), by field conditions, and, for maize, by the plant developmental stage (Renoud et al., 2020) as well as by soil depth (Gebauer et al., 2021). What is missing so far, is an analysis of the impact of water deficit on *acdS*^+^ communities under different farming systems. The α-diversity of microbial communities in organic farming systems is often described to be higher than that in conventional farming systems (Hartmann et al., 2015; Lupatini et al., 2017; Harkes et al., 2019), which increases the potential of the PGPR community to support plant growth in this land-use system (Hole et al., 2005; Jangid et al., 2008; Gomiero et al., 2011). Enriching *acdS*^+^ microorganisms in the rhizosphere represents an efficient means of stimulating plant growth during abiotic stress (Glick, 2014). Thus, an investigation of how the history of water deficit influences *acdS*^+^ gene community abundance and composition, and how this is related to agricultural management may provide essential insights into the potential of *acdS*^+^ soil bacteria to maintain crop production in the context of global change.

**Figure 1:**
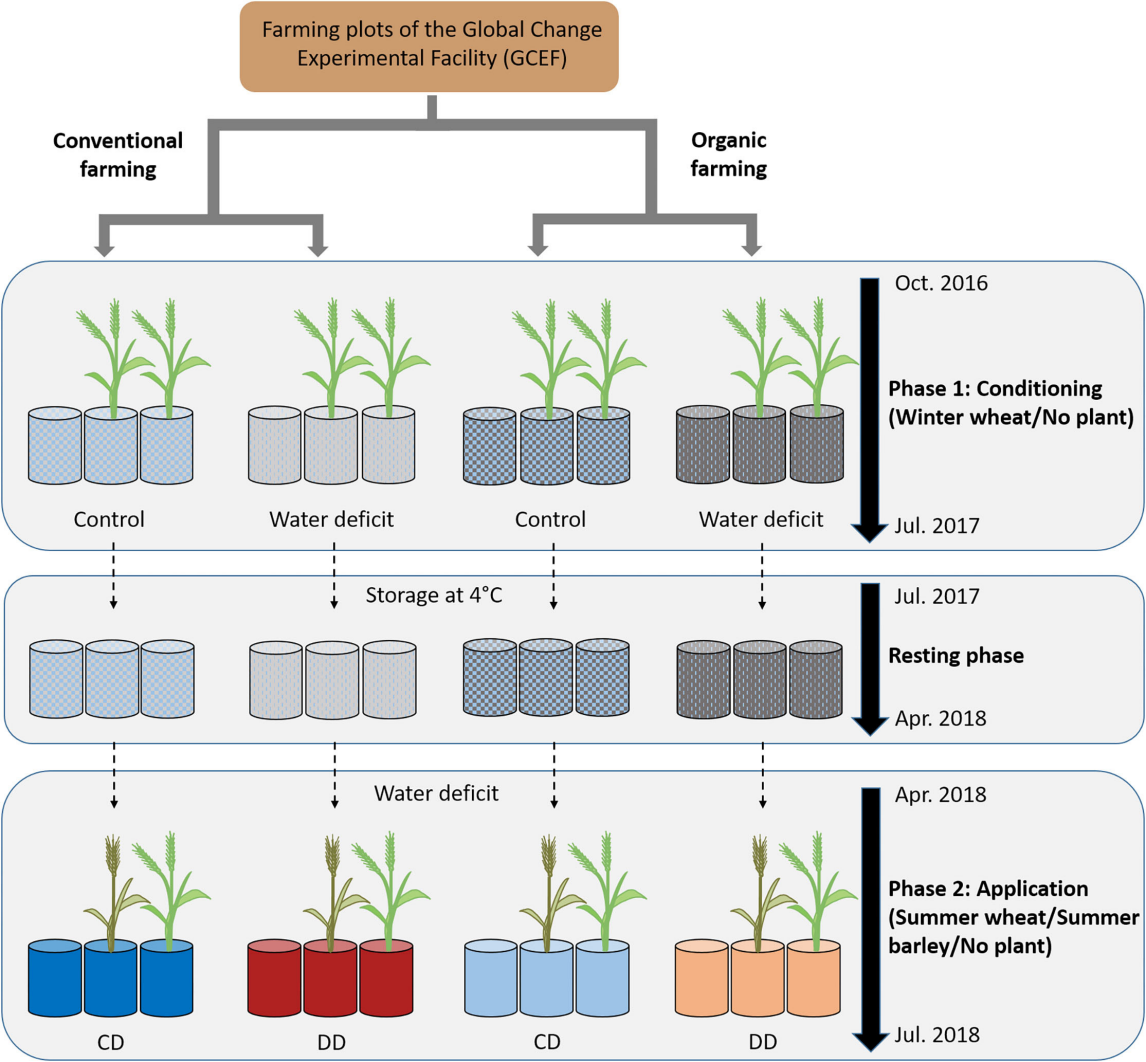
Experimental design. During the conditioning phase (October 2016 to July 2017), pots were filled with soil from the Global Change Experimental Facility (GCEF) managed under conventional or organic farming. Approimately two-thirds of the pots were planted with winter wheat, and one-third remained unplanted. Half of the pots were well-watered (60% maximum soil water content; WHC) while the other half were exposed to water deficit conditions (25% WHC), and appropriate fertilizer was applied for OF and CF (Breitkreuz et al., 2021). Plants were removed in July 2017, and the soil remained in the respective pots till April 2018 at 4°C. The application started in April to July 2018, with a water deficit (25% WHC) applied to all pots, and no further fertilization was applied. The pots planted with winter wheat during the conditioning phase were used to grow summer wheat or summer barley, while the unplanted pots remained with bare soil (*n* = 5 pots). CD: “single water deficit,” control and dry conditions, DD: “water deficit history,” dry conditions and dry conditions.

With respect to earlier studies on *acdS* gene markers, and since drought history experiments have shown that legacy effects interact with abiotic and biotic drivers (Kaisermann et al., 2017; Bennett and Klironomos, 2019; Canarini et al., 2021), we investigated here how the presence of cereals, cereal species, and farming systems modulate the legacy effect of water deficit on the *acdS*^+^ microbial community. To this end, we set up a pot experiment using Chernozem soil collected at conventional and organic farming plots of the experimental platform Global Change Experimental Facility (GCEF; Schädler et al., 2019) (Figure 1). Wheat (*Triticum aestivum* L.), as the first, and diploid barley (*Hordeum vulgare* L.) as the fifth most extensively cultivated cereal crop worldwide (www.fao.org/faostat), were chosen as model systems. The aim of the current study was to examine the impact of soil water deficit history, i.e., history of water scarcity, on the microbial community composition of wheat and barley grown under water deficit in a second year. For this approach, we analyzed both (i) the total prokaryote community using 16S rRNA gene amplicon sequencing (Illumina MiSeq) as well as (ii) the abundance and community composition of the functional group of *acdS*^+^ microorganisms (qPCR and Illumina MiSeq).

This work was based on three hypotheses. We first hypothesized that the presence of plants stimulates the selection of microbial communities and was further driven by water deficit history. We expected positive PSF in con-specific soil (wheat in the first and the second year of the experiment), assuming that with the same plant, the microbial community is already selected to dry conditions and niches in the rhizosphere that were present during the first period of water deficit will also be present during the second period of water deficit. Our second hypothesis was raised by the fact that bacterial diversity is expected to be higher under organic farming that represents limited resources but provides more niches (Thakur et al., 2020) and a broader functional pool of soil microorganisms to cope with drought stress. It stated that water deficit history modifies the microbial community assembly and that this effect is stronger in organic farming. Last, the third hypothesis was raised by reports on plant beneficial community selection by the rhizosphere (Mendes et al., 2014; Yuan et al., 2018). We hypothesized that the interaction between the history of water deficit and farming systems particularly impacts the *acdS*^+^ community composition in the rhizosphere and selects taxa adapted to dry conditions.

## Materials and Methods

### Soil Collection and Experimental Design

The pot experiment was conducted over two complete vegetation periods, a conditioning phase in the first year and an application phase in the second year (Figure 1). The experimental setup of the conditioning phase has been published in Breitkreuz et al. (2021). Briefly, the soil was collected from the upper 15 cm of organic (OF) and conventional (CF) farming plots of the Global Change Experimental Facility (GCEF, Schädler et al., 2019) at the UFZ field research station in Bad Lauchstädt, Central Germany [51°2393599N 11°5295599E, 118 m above sea level, average annual temperature: 9.7°C (1993 to 2013)]. The soil type at the research station is a fertile, loamy Haplic Chernozem (Altermann et al., 2005). The organic and conventional farming systems were implemented in autumn 2013. The conventional management comprises a rapeseed-wheat-barley crop rotation and the application of mineral NPK fertilizer, pesticides, and plant growth regulators. In contrast, organic management is conducted according to the EU regulation for organic agriculture (European Union, 2007), i.e., without applying plant protection or growth products. Moreover, mineral N fertilizer is replaced by the inclusion of legumes in the crop rotation (legume-wheat-barley). Besides the biological nitrogen fixation *via* legumes, fertilizers in the organic management are exclusively applied as rock phosphate and patentkali (K-Mg-S). The pre-adapted soils were homogenized and sieved (10 mm). For the pot experiment, 9.3 kg Chernozem was weighed in bags, mixed with fertilizers and water (60% of soil type-specific maximum water holding capacity; WHC), and filled in 7 L Kick–Brauckmann pots (STOMA GmbH, Siegburg, Germany). The applied fertilizers were selected in accordance with the guidelines for conventional and organic farming. Phosphorus (P), potassium (K), and nitrogen (N) sources for conventional farming were triple super phosphate, 60 % K_2_O (60er Kali), and ammonium nitrate, while for organic farming they were granulated raw phosphate (Physalg 25), Muriate of potash (patentkali), and urea, respectively. Both conventional and organic farming pots were equally fertilized with 2 g N, 1 g P, and 2 g K. Further addition of 0.5 g Mg (MgSO_4_), 0.15 g FeCl_3_, and a mixture of micronutrients (A–Z solution by Hoagland and Snyder, 1933) was exclusive for conventional farming pots.

The pots were first subjected to a conditioning phase, comprising a four-factorial-design (Breitkreuz et al., 2021). Briefly, the pots were filled with soil, which originated from field experiments in Thyrow (Albic Luvisola, sandy soil) and Bad Lauchstädt (Haplic Chernozem, fertile soil) and was long-term adapted to either conventional or organic farming systems. We further decided on two different winter wheat genotypes with different site specifications to grow (demanding vs. non-demanding) and compared them to unplanted pots as control. Winter wheat was sown with either fungicide-treated seeds per pot for the conventional farming treatment or 16 untreated seeds per pot for the organic farming treatment. The pots were randomly placed on trolleys in a cold greenhouse and kept at 60% WHC over the winter of 2016/17. To guarantee germination, seeds were initially watered from the top until the plants reached the three-leaf stage. In March 2017, plants were equally adjusted to a number of 12 per pot and the watering treatment started. For this purpose, half of the pots were set to 25% WHC. The final design was thus: 2 soil types × 2 farming systems × 3 plant treatments × 2 watering systems x 5 replicates of each treatment = 120 pots. The water content was controlled daily by weighing the pots and adding the lost water. We observed that the prokaryotic communities were comparable between the two wheat cultivars (Breitkreuz et al., 2021). After the harvest of wheat plants and bare soil in July 2017, the pots were kept at 4°C to preserve the adapted microbial communities. We expected that the microbial communities would remain stable during the storage in the cold room, but we are aware that to be absolutely sure about this, an analysis of pots before and after the storage would have been important to address this issue.

In April 2018, the application phase started. No further fertilization was applied to preserve adapted communities from the conditioning phase. Summer wheat (“Quintus,” approved 18th December 2013, W. von Borries-Eckendorf GmbH & Co. KG, Germany) and summer barley (“Avalon,” approved 20th December 2012, Saatzucht Josef Breun GmbH & Co. KG, Germany) were sown (12 plants per pot) in the pre-adapted pots from the conditioning phase (Figure 1). Both plants species are characterized by broad acceptance of cultivation sites and moderate resistance to drought. The pots were randomly placed on trolleys in a cold greenhouse. To guarantee germination, the seeds were initially watered from the top until the plants reached the three-leaf stage. Thereafter, all pots were subjected to dry conditions (25% WHC), and water loss was compensated daily from the bottom. Shortly before sampling, during the flowering stage in June 2018, above-ground plant heights were determined.

Three plants per pot were harvested to obtain a replicate. Fresh and dried (at 60°C) above-ground biomass was recorded. The soil that was closely attached to the roots, considered the rhizosphere, was carefully collected by brushing, frozen in liquid nitrogen, and stored at −80°C (rhizosphere “wheat” or “barley”). From each pot without plants (“bare soil”), three soil cores were pooled, sieved to 2 mm, frozen in liquid nitrogen, and stored at −80 °C. A total of five replicates (each composed of the three pooled rhizosphere or core soils) were analyzed in this study for each treatment of the application phase. The application phase treatments were termed after watering treatments applied in the first and second year: CD (“single water deficit,” control and dry conditions) and DD (“water deficit history,” dry conditions and dry conditions).

### Construction of 16S rRNA Gene and *acdS* Libraries and Sequencing

To analyze the microbial community composition of the samples, DNA of 400 mg soil was extracted (*n* = 5 for each treatment) with the DNeasy PowerSoil kit (QIAGEN, Hilden, Germany) according to the manufacturer's instructions. DNA purity and quantity were measured with a NanoDrop (ThermoFisher Scientific, Waltham, MA, USA) and extracts stored at −20°C. The amplification of the bacterial 16S rRNA gene V4 region was performed with the universal primer pair 515f and 806r (Caporaso et al., 2011), and the partial *acdS* gene was amplified using the primers *acdS*F5 and *acdS*R8 (Bouffaud et al., 2018), both in duplicate. Primers were equipped with Illumina adapter sequences (Nextera XT Index Kit, Illumina, San Diego, CA, USA). To obtain high-fidelity amplification, PCR was performed using Kapa Hifi HotStart ReadyMix (KAPA-Biosystems, Wilmington, MA, USA). The PCR was performed in a S1000 Thermal Cycler (Bio-Rad Laboratories, Hercules, CA, USA): Initial denaturation at 95°C for 5 min, followed by 25 cycles of 98°C for 20 s, 55°C for 15 s, 72°C for 15 s−16S rRNA gene/30 cycles of 65°C for 10 s, 72 °C for 10 s—*acdS*, and final elongation at 72°C for 5 min. PCR products were purified using AMPure XP beads. To assign the sequences to the respective samples, an index PCR was performed using the Illumina Nextera XT Index Kit and Kapa Hifi HotStart ReadyMix (KAPA Biosystems, Wilmington, MA, USA). The indexed PCR was performed in an S1000 Thermal Cycler (Bio-Rad Laboratories, Hercules, CA, USA): 8 cycles of 95°C for 30 s, 55°C for 30 s, and 72°C for 30 s. PCR products were again purified with AMPure XP beads and quantified with Quant-iT PicoGreen dsDNA Assay Kit (Invitrogen, Life Technologies, Carlsbad, CA, USA) following the manufacturer's instructions. For sequencing, samples were pooled, and the pool's size and quality were checked with an Agilent 2100 Bioanalyzer (Agilent Technologies, Palo Alto, CA, United States). Paired-end sequencing was performed on 16S rRNA and *acd*S libraries using the Illumina MiSeq system. Raw sequences are accessible in the Short Reads Archive under the Bioproject PRJNA783187.

### Sequence Data Processing

For both amplicon datasets, only reads with the expected amplification primers were kept, and reads without these primers were removed from further analysis. Primer sequences of the 16S rRNA gene amplicons were removed using cutadapt version 1.18. The obtained sequences were analyzed using dadasnake version.7 (Weißbecker et al., 2020; https://github.com/a-h-b/dadasnake) which depends on the open-source program R's (version 3.6.1; R Core Team 2017) DADA2 package (Callahan et al., 2016). The 16S rRNA gene amplicon reads were truncated to a minimum base quality of 11 and overall maximum expected error of 5, with a minimum length of 150 and 100 nt of the forward and reverse reads. The shorter *acdS* amplicon reads were truncated to 100 and 90 nt for the forward and reverse reads with a minimum base quality of 11 and a maximum expected error of 0.7. For both genes, read pairs were merged with zero mismatches, and exact sequence variants were determined to be used as ASVs (Amplicon Sequence Variants). Chimeric reads were removed using the DADA2 “consensus” algorithm (Callahan et al., 2016). Subsequently, the 16S rRNA gene amplicon sequence variants were taxonomically assigned using the mothur implementation of the ‘Bayesian Classifier' (Schloss et al., 2009) against the SILVA database (version 132, non-redundant at 99%; Quast et al., 2013). The *acdS* sequences were aligned against an in-house *acdS* database extracted from FunGene (Fish et al., 2013) using BLASTn (version 2.7.1), according to Bouffaud et al. (2018). The 16S rRNA gene amplicons ASVs that were not assigned to the kingdoms Bacteria or Archaea were removed, and for the *acdS* amplicons, ASVs not assigned taxonomically using BLASTn were also removed.

### Real-Time Quantitative PCR

The 16S rRNA and *acdS* genes were quantified in all samples using primers with Illumina linkers for 16S rRNA genes and linker-free *acdSF5/acdSR8* primers for *acdS* genes (Bouffaud et al., 2018). For real-time PCR master mix was prepared to contain 0.5 μl of each primer with a concentration of 10 μM and ~20 ng of DNA and made up to a final volume of 15 μl with 2x iQ SYBR Green Supermix. Amplification was run in the iCycler iQ5 (Bio-Rad Laboratories) with the following program: 45 cycles of 94°C for 15 s, 67°C for 15 s, 72°C for 15 s−16S rRNA gene/67°C for 15 s, 72°C for 15 s–*acdS*. The amount of *acdS* genes was normalized by the 16S rRNA gene copy numbers according to the comparative method of Livak and Schmittgen (2001).

### Statistics

All statistical analyses and visualizations were performed in R (version 3.6.1; R Core Team, 2017). Fresh and dry plant weights were compared using the Kruskal–Wallis test followed by Dunn's test, as the data were not normally distributed. The effects of the different treatments on plant height as well as on the relative gene expression from qPCR were analyzed using ANOVA followed by Tukey's HSD test. Since the relative gene expression datasets were partly highly skewed, in addition to ANOVA and Tukey tests, the Kruskal–Wallis test followed by *post-hoc* tests were carried out using the Fisher's least significant difference criterion and Benjamini-Hochberg correction in “agricolae” package. Shannon index was calculated for each sample using the “vegan” package and the effect of the treatments on the microbial diversity was tested by the Kruskal–Wallis test followed by Fisher's LSD *post-hoc* test with Benjamini-Hochberg adjustment. ASV patterns were cross-compared with permutational multivariate analysis of variance (PERMANOVA; Anderson, 2001) using the “vegan” package. The effects of the plant, conditioning, and farming on the dominant taxa were tested by PERMANOVA. The effect of water deficit history on differential abundance was tested using DESeq2 for the ASVs representing at least 0.05% of the unrarefied reads (Love et al., 2014), comparing the farming system and the factor plant independently. Correlations between *acdS* and 16S rRNA gene read numbers were analyzed by Spearman's rank correlation test.

## Results

### Plant Growth

**Figure 2:**
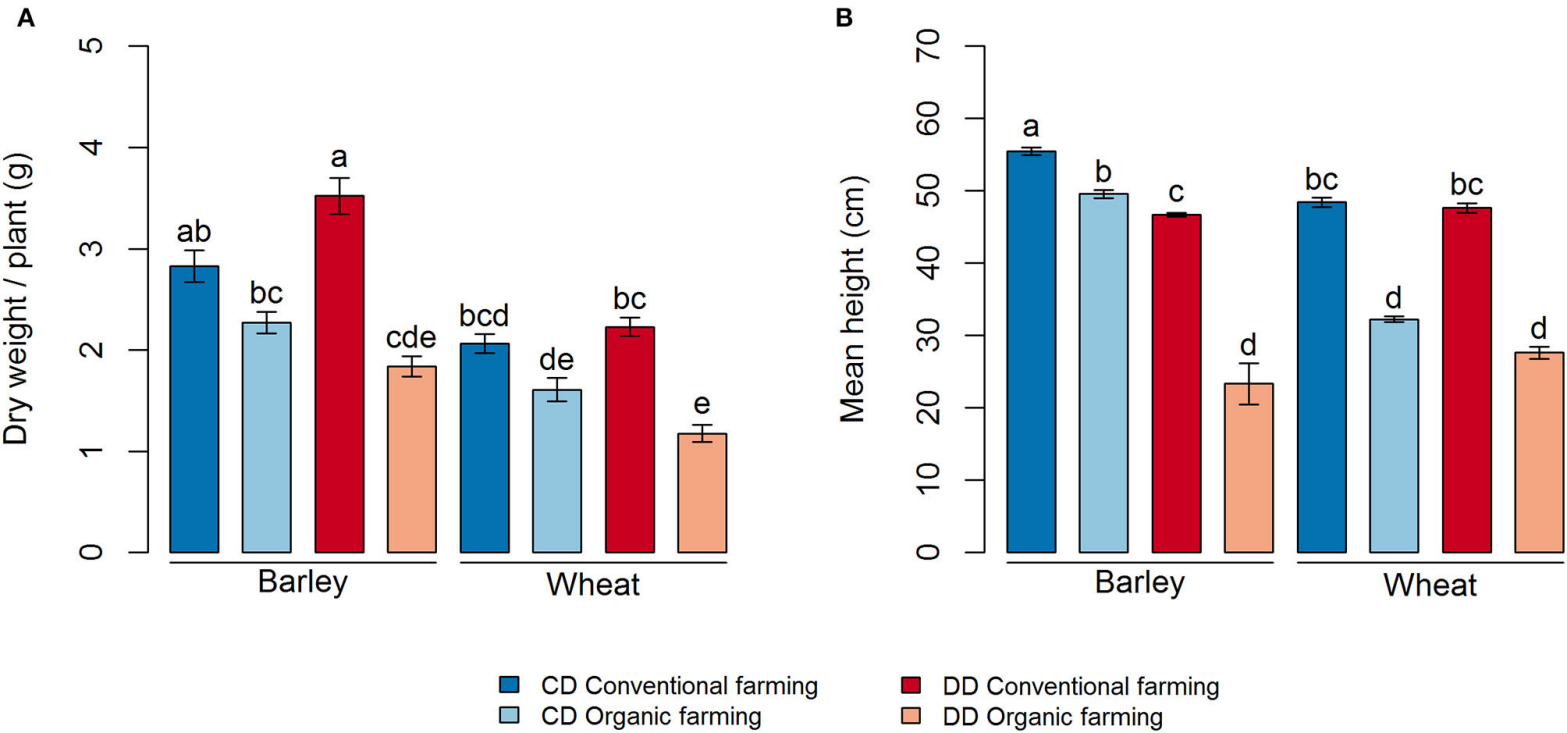
Plant growth measures for each treatment, **(A)** dry weight per plant and **(B)** mean height. Values correspond to the average, and error bars to the respective standard error, of five pot, replicates. Letters show the statistical differences between treatments according to the Kruskal–Wallis test followed by Fisher's LSD *post-hoc* test with Benjamini-Hochberg *P*-value adjustment. CD, no water deficit conditioning; DD, water deficit conditioning.

Plant growth was strongly affected by farming management (CF > OF, *P* < 0.001 for both dry weight and height) (Figure 2). Thereby, the biomass of both portions of cereal was lower under organic (OF) than under conventional farming (CF), but this difference was only significant in the DD treatment (Figure 2A, *P* < 0.001 and *P* = 0.003, for barley and wheat, respectively). In addition, plant height was higher in CF than in OF for both crops in DD and CD (Figure 2B). The effect of water deficit conditioning (DD vs. CD) on the plant parameter was limited to a negative impact of water deficit conditioning on the height of the barley plants, in both OF and CF (*P* < 0.001, Figure 2B). Interestingly, water deficit conditioning tended to have a positive, but not significant, effect on barley dry weight under CF.

### Relative Abundance of *acdS*-Carrying (*acdS*^+^) Microorganisms

**Figure 3:**
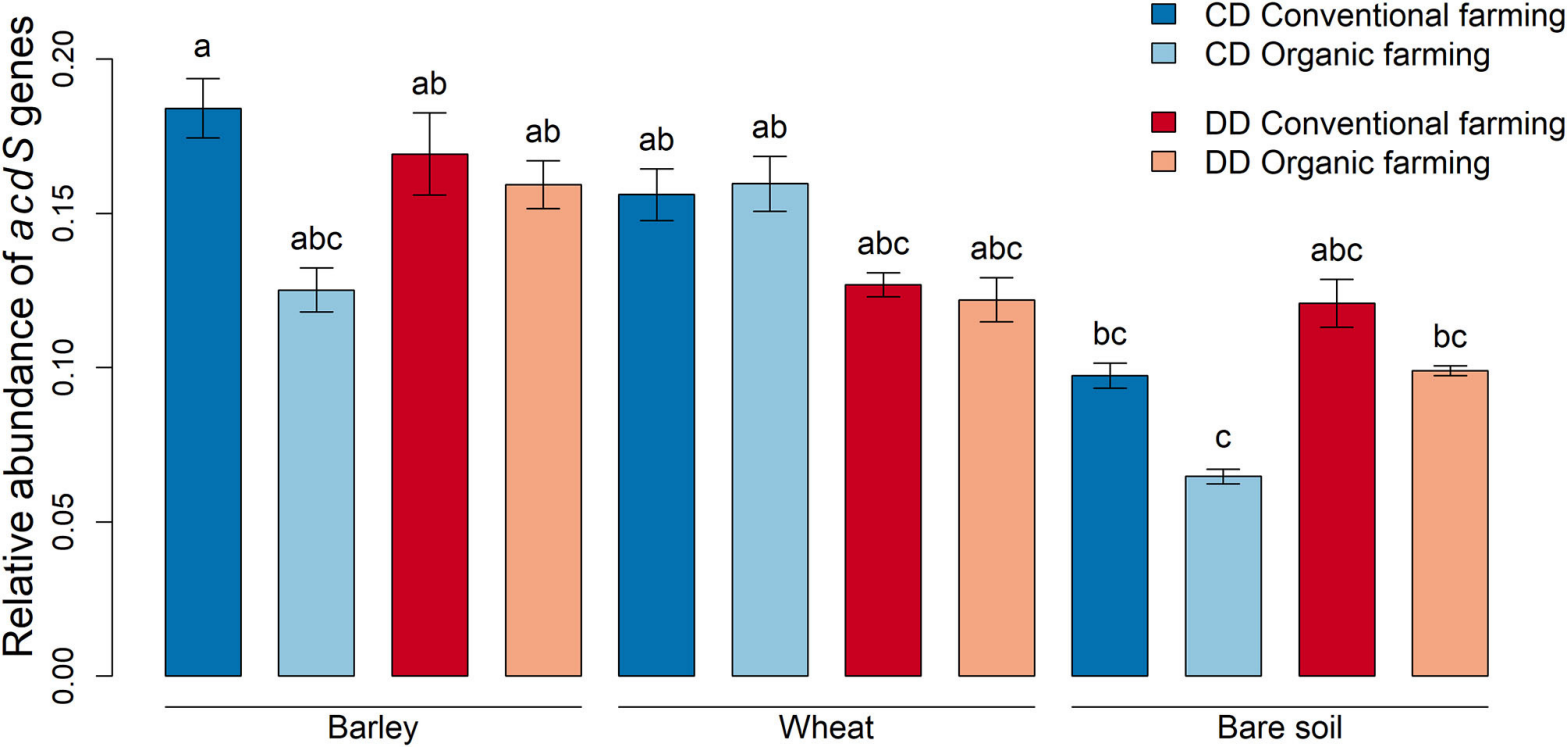
Relative abundances of microorganisms with genes encoding for ACC deaminase. Abundance data obtained by qPCR were normalized with 16S rRNA gene abundances and calculated using the ΔCt method (Livak and Schmittgen, 2001). Values correspond to the average, and error bars to the respective standard error, with five pot, replicates. Letters show the statistical differences between all treatments according to ANOVA followed by Tukey's HSD *post-hoc* test. CD: “single water deficit,” control and dry conditions, DD: “water deficit history,” dry conditions and dry conditions.

To investigate whether the relative abundance of *acdS*^+^ microorganisms was affected by the treatments, the amplification levels of *acdS* genes were normalized with the abundances of 16S rRNA genes using the ΔCt method (Livak and Schmittgen, 2001). When all rhizosphere samples (without differentiating by plant species, farming system, or water conditioning) were compared to the bare soil samples, we observed that *acdS* genes were less abundant in bare soil than in rhizosphere soil (*P* < 0.001). More precisely, this difference was significant in CD under CF for barley (*P* = 0.03) and under OF for wheat (*P* = 0.006, Figure 3). When the effect of water conditioning on the relative abundance of *acdS* genes was tested for the plant rhizosphere samples, no significant difference was found between CD and DD. However, a significant positive effect of the water conditioning on the relative abundance of the *acdS*^+^ community was found in the bare soil samples (*P* = 0.02).

### Effects of Water Deficit History, Land Use, and Plant Presence on Bacterial Community Composition and Diversity

Barley and wheat rhizospheres, as well as bare soil samples, were subjected to 16S rRNA and *acdS* gene amplicon sequencing. In total, 2,225,931 16S rRNA and 4,406,511 *acdS* sequencing reads formed 1,849 ASVs and 4,945 ASVs, respectively. The rarefaction curves are presented in Supplementary Figure S1. The sequences were rarefied to the smallest read numbers per sample (16S rRNA gene 20,058 reads; *acdS* gene 54,660 reads). Altogether, the 16S rRNA gene reads covered a broad phylogenetic range, and the 10 most abundant classes corresponded to Actinobacteria, Alphaproteobacteria, Gammaproteobacteria, Thermoleophilia, Chloroflexi, Gemmatimonadetes, Bacteroidia, Blastocatellia, Nitrososphaeria, and Verrucomicrobiae. By contrast, the *acdS* amplicons were largely associated with only two phyla, Actinobacteria, and Proteobacteria. The majority, 79.6% of the rarefied reads, were affiliated with Actinobacteria, mainly of the families Streptomycetaceae, Intrasporangiaceae, and Nocardioidaceae. Almost a fifth of the *acdS* reads (19.6%) corresponded to Proteobacteria. While 0.8% were unclassified (u.) bacteria, we also gathered 0.0014% corresponding to fungi in the Ascomycota. In order to verify if the changes in the total bacterial community between the treatments were also visible at the *acdS* community level, all genera from the *acdS* and 16S rRNA gene communities were compared with each other. In total, 32 genera were identified in both communities, 18 belonging to the Actinobacteria and 14 to the Proteobacteria. Significant correlations between the distribution patterns of 16S rRNA and *acdS* gene reads were found for five genera identified from the very abundant ones, *Saccharothrix, Amycolatopsis, Marmoricola, Tetrasphaera*, and *Streptomyces* (Supplementary Figure S2), as well as *Achromobacter (P* = 0.021, rho 0.3) and u. *Microbacteriaceae* (*P* = 0.006, rho −0.36).

Permutational analysis of variance indicated that plant presence was the main factor driving both 16S rRNA (*R*^2^ = 0.36, *P* < 0.001) and *acdS* (*R*^2^ = 0.22, *P* < 0.001) community composition (Table 1). Moreover, Shannon diversity indices of the bacterial (Supplementary Figure S3a) and *acdS* communities (Supplementary Figure S3b) were overall higher in bare soil than in rhizosphere samples. This difference was detected for both targeted genes and for all treatments. Although significant, the explanatory values for the effects of conditioning (CD or DD, 16S rRNA: *R*^2^ = 0.07, *P* < 0.001; *acdS*: *R*^2^ = 0.04, *P* = 0.002) and farming system (16S rRNA: *R*^2^ = 0.05, *P* < 0.001; *acdS*: *R*^2^ = 0.05, *P* < 0.001) on community composition were comparatively low. In total, 60% of the total variance in the total prokaryotic, and 46% of the variance in the *acdS*^+^ community composition could be explained by the sole effects of plant presence, water deficit conditioning, and farming alone or by its interaction.

**Table 1:** PERMANOVA of the 16S rRNA and *acdS* gene composition.

		**All treatments (including bare soil)**		**Wheat and barley treatments**	
		***R*** **^2^**	***P*** **-value**	***R*** **^2^**	***P*** **-value**
16S rRNA gene					
	Plant	**0.36**	**<0.001**	0.03	0.067
	Farming	**0.05**	**<0.001**	**0.11**	**<0.001**
	Conditioning	**0.07**	**<0.001**	**0.17**	**<0.001**
	Farming × conditioning	0.01	0.112	**0.04**	**0.044**
	Farming × plant	**0.04**	**0.035**	0.02	0.342
	Conditioning × plant	**0.05**	**0.004**	0.02	0.526
	Farming × conditioning × plant	0.02	0.253	0.02	0.652
	Residual	0.40		0.60	
*acdS*				
	Plant	**0.22**	**<0.001**	**0.05**	**0.002**
	Farming	**0.05**	**<0.001**	**0.11**	**<0.001**
	Conditioning	**0.04**	**0.002**	**0.07**	**<0.001**
	Farming × conditioning	**0.02**	**0.044**	**0.05**	**0.008**
	Farming × plant	**0.05**	**0.011**	0.03	0.07
	Conditioning × plant	**0.05**	**0.009**	0.03	0.098
	Farming × conditioning × plant	0.03	0.088	0.02	0.405
	Residual	0.54		0.63	

*The ASV composition was considered for all treatments, to determine the effect of plant (wheat, barley, and bare soil), farming system (organic and conventional farming), and watering condition during the conditioning phase (CD and DD). The additional analysis of only barley and wheat treatments was used to analyze the effect of the plant species on the 16S rRNA gene base and acdS^+^ community compositions. Significant factors (P < 0.05) are indicated in bold*.

**Figure 4:**
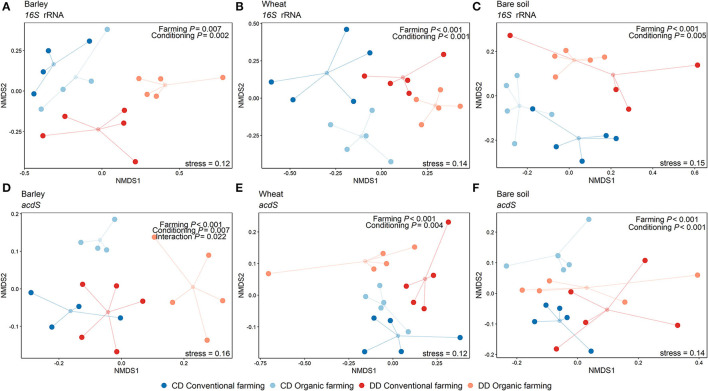
Non-metric multidimensional scaling showing the effects of the farming system and watering during the conditioning phase per plant treatment. NMDS was performed on Amplicon Sequence Variants (ASV) using the Bray-Curtis dissimilarity matrix. The small circles correspond to the geometric center (centroid) of each treatment, and the lines indicate the distance of the samples to the centroid. Data from the 16S rRNA gene-based analysis are shown in subfigures a-c and *acdS* gene analyses are presented in subfigures d-f, for barley **(A,D)**, wheat **(B,E)**, and the pots without plants **(C,F)**. Results of the PERMANOVA are indicated in each plot (*n* = 5 pots). CF: conventional farming, OF: organic farming, CD: “single water deficit,” control and dry conditions, DD: “water deficit history,” dry conditions and dry conditions.

Since the strong difference between the absence and the presence of plants masked the potential effects of plant identity, the rhizosphere samples were again analyzed separately from the bare soil ones. In this subset, the three factors explained 40% of the total variance in the 16S rRNA and 37% of the total variance in the *acdS*^+^ community composition. Plant species identity (barley or wheat) had no impact on the total bacterial community composition, but a significant effect on *acdS*^+^ community composition (*R*^2^ = 0.05, *P* = 0.002). Furthermore, the farming system (*R*^2^ = 0.11, *P* < 0.001) and water conditioning (*R*^2^ = 0.07, *P* < 0.001), as well as the interaction between these factors, significantly shaped *acdS* community composition (Table 1), in particular for barley (Figure 4). A similar pattern was found for 16S rRNA gene composition, whereby conditioning (*R*^2^ = 0.17, *P* < 0.001) and farming system (*R*^2^ = 0.11, *P* < 0.001) were the main drivers (Figure 4). Crop-dependent effects were also found for Shannon indices (Supplementary Figure S3). Thus, for barley, the lowest diversity indices comparing all treatments were found for both *acdS* and 16S rRNA genes in OF under DD (*P* = 0.004 and *P* = 0.032 for *acdS* and 16S rRNA genes, respectively). For wheat, only the Shannon index of 16S rRNA genes was negatively impacted by the history of water deficit under OF, with a lower index in DD than in CD (*P* = 0.015). In contrast, the Shannon index of the *acdS* community in the rhizosphere of wheat was positively influenced by water deficit history under CF (DD > CD, *P* = 0.02) and further by farming soil type in DD (CF > OF, *P* < 0.001).

### Distribution of Dominant Bacterial Genera Across Treatments

The 20 most abundant genera identified in the wheat and barley rhizospheres, as well as in the bare soil samples contributed on average 52.1 % to the total 16S rRNA gene amplicon reads (Supplementary Figure S4). Out of these, one genus was assigned to Archaea and 19 genera to Bacteria. Within the bacteria, the phylum of Actinobacteria was represented by six genera and Proteobacteria by five genera. Other phyla comprised each of two genera of Gemmatimonadetes (*Gemmatimonas* and u. Gemmatimonadaceae), Acidobacteria (*Acidobacteria SG6* and *RB41*), and *Chloroflexi* (*JG30 KF CM45* and *KD4 96*), and one genus of *Verrucomicrobia* (*C. Udeobacter*) and *Bacteroidetes* (u. Chitinophagaceae), respectively. The relative abundances of the 20 most abundant 16S rRNA gene-based genera varied among treatments (Supplementary Table S1). As for the whole community, three-way ANOVA performed on each of the 20 most abundant 16S rRNA gene-based genera showed that the presence/identity of the plant and then the farming and conditioning significantly shaped their distribution (Supplementary Table S1, Supplementary Figure S4). Genera from u. Chitinophagaceae, *Gemmatimonas*, and *Acidobacteria subgroup 6* were only affected by the plant presence but not by the farming system or the conditioning. For both plant rhizosphere samples, a farming effect was found for *Rhodanobacter* (OF > CF) and *Glycomyces* (CF > OF), and a conditioning effect for C. *udeobacter* and Chloroflexi *JG30-KF-CM45* (CD > DD) as well as for *Dyella* and *Nocardioides* (DD > CD). Finally, a farming and a conditioning effect were present for both plant rhizospheres, with a prevalence of u. Nitrososphaeraceae, KD4 96 and u. Solirubrobacterales in the CF and CD treatments and *Sphingomonas* and *Streptomyces* in the OF and DD treatments (Supplementary Table S1).

**Figure 5:**
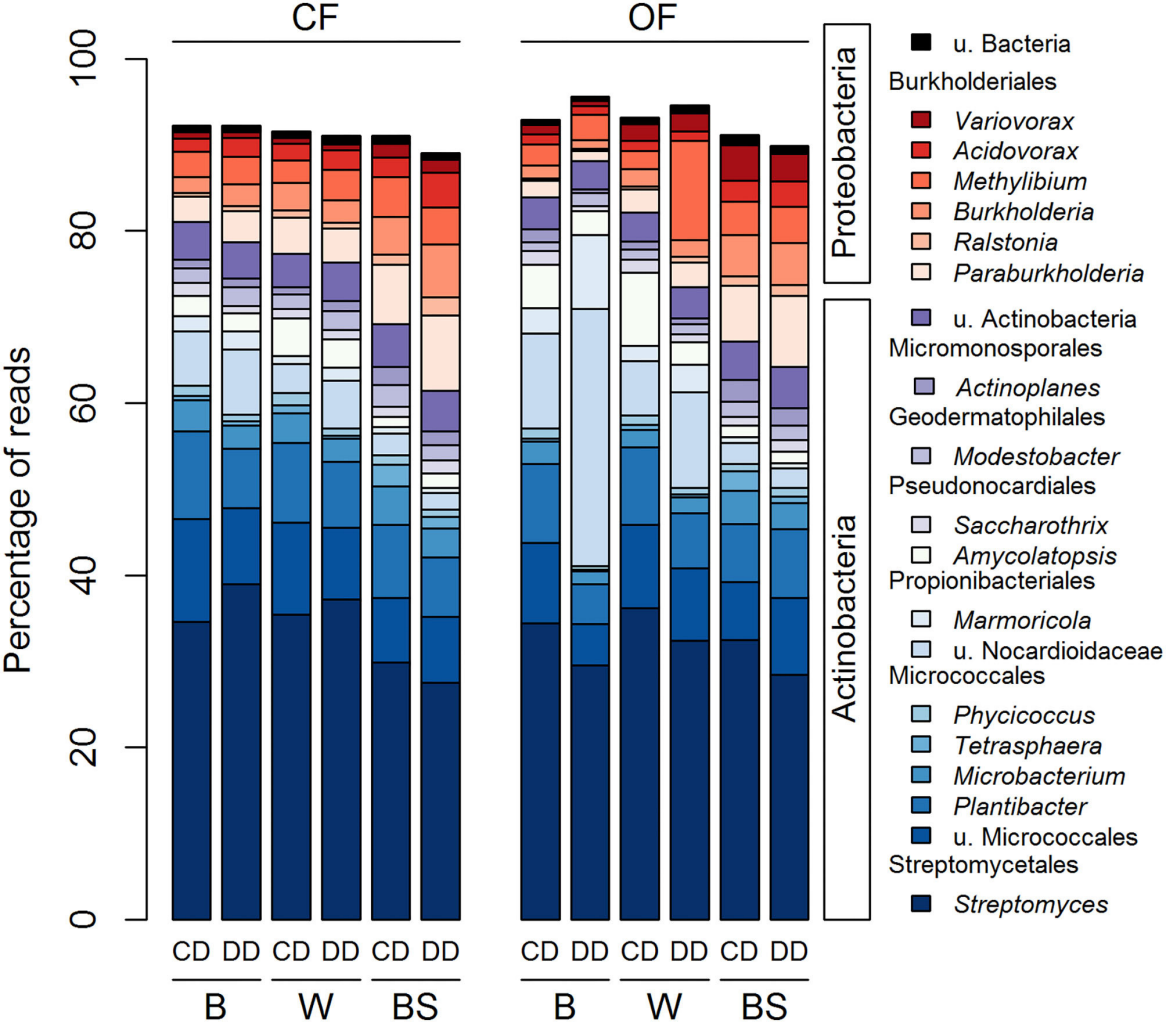
Distribution of the 20 most abundant genera across all treatments based on the *acdS* dataset. CF, conventional farming; OF, organic farming; B, barley; W, wheat; BS, bare soil; CD, “single water deficit,” control and dry conditions; DD, “water deficit history,” dry conditions and dry conditions; *n* = 5 pots.

The *acdS* sequences from the 20 most abundant genera in the rhizosphere and bare soil represented on average 92% of the total number of reads (Figure 5). The sequences were either affiliated with the Actinobacteria (79.6 % of total reads) or the Proteobacteria (19.6 % of total reads). Actinobacteria were represented by 13 genera distributed among five different orders: one genus from the Streptomycetales, five genera from the Micrococcales, two from the Propionibacteriales, two from the Pseudonocardiales, one from the Geodermatophilales and from the Micromonosporales and the group of u. Actinobacteria. The seven genera belonging to the Proteobacteria corresponded to six genera of the order Burkholderiales and one of u. Bacteria. Three-way ANOVA analyses showed that 19 out of the 20 most abundant *acdS* genera were significantly affected by at least one factor (Figure 5 and Supplementary Table S1), with the exception of the *Burkholderia* (Proteobacteria). These abundant genera were strongly affected by the plant presence, but also by the farming system and the conditioning, or their combined effects. In contrast to the 16S rRNA gene-based analysis, only two genera were only impacted by the plant presence without the direct effect of farming or conditioning. The other 18 genera were impacted in the plant rhizosphere by the effect of the farming system (five genera), conditioning (four genera), or by both (eight genera) (Supplementary Table S1).

### Water Deficit History Responsive Genera of the *acdS*+ Community in the Rhizosphere

Due to the higher effect of water conditioning on the *acdS*+ bacteria in the rhizosphere (Table 1), their higher relative abundance in the rhizosphere compared to the bulk soil (*P* < 0.001) and the hypothesis that this functional group of bacteria helps the plant to face stress conditions, the bare soil was not analyzed to determine the effect of water deficit conditioning. The abundances of the unrarefied rhizosphere ASVs representing at least 0.05% of the *acdS*^+^ reads corresponding to 377 ASVs with at least 1,400 reads were determined, and the log_2_ fold changes between CD and DD were calculated (Supplementary Table S2).

**Figure 6:**
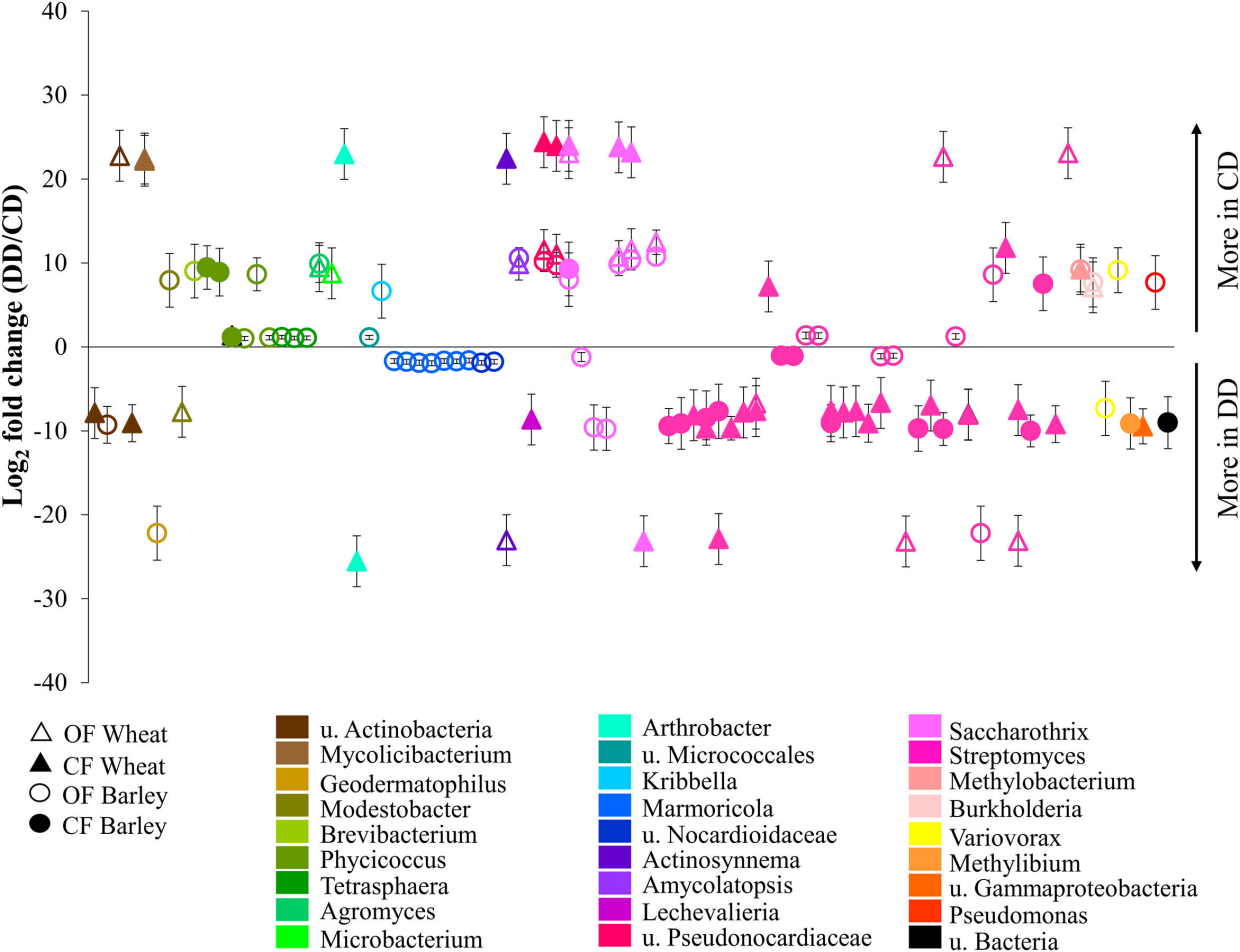
Relative abundances of the *acdS*^+^ ASVs significantly impacted by drought conditioning. Taxa were identified using DESeq2 and *P*-value < 0.05, and log2-fold changes between respective treatments are represented. CF, conventional farming; OF, organic farming; CD, “single water deficit,” control and dry conditions; DD, “water deficit history,” dry conditions and dry conditions; *n* = 5 pots.

For both plants and under both farming systems, 122 conditioning-responsive ASVs were detected (Supplementary Table S2), including 87 with a fold change higher than two (Figure 6). The results showed that the effect of conditioning on the abundance of *acdS*^+^ ASVs was often planting species- and farming system-specific (Figure 6). Much higher abundances of ASVs under water deficit conditioning (DD) treatment (log2 fold change < −12) were highly associated with samples from CF, especially ASVs associated with Actinobacteria. Interestingly, a significantly higher abundance of several ASVs corresponding to *Marmoricola* sp. were only found under OF. In addition, some Actinobacteria ASVs were only detected under DD (log2 fold change < −20) and this pattern was found independently of the farming condition. In contrast, the ASVs detected only in the treatments without drought conditioning (CD, log2 fold change > 20) were exclusively found in the wheat treatments, independently of the farming condition.

## Discussion

Climate predictions suggest an increased probability of summer drought in Central Europe (Spinoni et al., 2018; Hari et al., 2020), which represents a threat to plant water and nutrient uptake. This threat is supposed to be mitigated by plant-beneficial rhizobacteria (de Vries et al., 2020). Drought legacy effects may have a strong effect on soil functioning, by long-term adaptations in the microbial community composition and microbial-mediated plant functioning. For the first time, this study analyzed the impact of successive dry conditions on the diversity and community composition of the *acdS*^+^ functional group of microbes potentially contributing to plant stress tolerance. We show that the rhizosphere prokaryotes and especially the *acdS*^+^ community are adaptable to water limitations. The impact of the watering regime during the application phase on the microbial communities indicates a legacy of water deficit.

### Water Deficit Conditioning Structures the Microbial Community, Especially in the Rhizosphere

We expected in our first hypothesis that the response to water deficit conditioning by the microbial communities would be specific for planted and non-planted pots due to the selection of specific microbes by the roots. Our results show that this proved to be the case. We observed that plant presence was the main factor influencing both 16S rRNA and *acdS* gene diversity, and this was reflected by distinct community compositions in bare soil and in the rhizosphere of both barley and wheat. We would like to point out that we did not perform an analysis of the soil outside the rhizosphere in the planted pots, which would have been an important supplementary control treatment. Due to the strong selection effect of plants in combination with adaptation to water stress conditions, the microbial community is supposed to be less complex in the rhizosphere soil (Mendes et al., 2014), which was confirmed by the lower diversity (Shannon index) of the total bacterial as well as the ACC deaminase microbial communities in the rhizosphere treatments compared to the bare soils. However, despite this reduced diversity, water deficit conditioning-induced community shifts were more obvious in the rhizosphere than in the bare soil. As root exudation changes under drought conditions (Preece and Peñuelas, 2016), these results suggest specialization of the taxa recruited in the rhizosphere in order to help the plant to face the stress.

### Farming Systems Select Taxa After Recurrent Drought Events

Soils under organic farming are often a reservoir of higher microbial diversity and activity than soils under conventional farming due to higher soil organic carbon levels (Lori et al., 2017; Harkes et al., 2019), which may lead to a stronger plant-beneficial response to stress. However, contrary to our second hypothesis, we found that several taxa were enriched in the plant rhizosphere under conventional farming after the water deficit conditioning. Similarly, while the diversity of the *acdS*^+^ community decreased in the rhizosphere under organic farming in DD, it increased in DD under conventional farming. Noteworthy, this higher diversity of plant-beneficial, *acdS*+ taxa under conventional farming was not visible at the level of the entire bacterial community, suggesting that plants under water deficit specifically selected plant beneficial organisms to counteract the stress.

These farming type-specific effects on microbial communities are in line with the indicator species that were identified from agricultural soils after short drought stress by Kundel et al. (2020). Under moderate drought, plants release more organic acids in their root exudates, especially the drought-tolerant ones (Song et al., 2012). Organic acids stimulate microbial activity in the rhizosphere (Macias-Benitez et al., 2020). In our samples under conventional farming, plants had a better fitness due to the fertilization amendments in CF, which may lead to higher exudation rates of these organic acids, and to the selection of the plant-beneficial ACC deaminase bacteria after a successive period of water deficit. Consequently, the *acdS*^+^ taxa specifically enriched in the rhizosphere after water deficit conditioning potentially represent good indicators of drought-adapted microbes.

### History of Water Deficit and Farming Systems Affect Strongly the *acdS*+ Community in the Rhizosphere

The majority of the dominant microbial taxa were influenced by water deficit conditioning, which may be surprising, as not all microbial individuals within a genus have similar functions, and the effect of a particular treatment might be diluted at a certain phylogenetic level. During the conditioning phase, the predicted abundance of ACC deaminase-related genes was higher in organic farming communities of well-watered soils compared to drought treatment (Breitkreuz et al., 2021). This difference in abundance was not observed in the DD samples of organic farming of barley or wheat in the present study. However, and presumably, as a result of this, the Shannon index was lower in organic farming DD, especially in the barley rhizosphere. This suggests that the *acdS*+ community was already under selection during the conditioning phase, especially by water limitation in organic farming, and some less abundant taxa might have been already selected and their proportions increased. Consequently, the selection during the conditioning phase may have led to a decrease in Shannon index at the application phase that was further enhanced by hetero-specific plant-soil feedback which means changing the plant from wheat in the conditioning phase to barley in the application phase.

In this study, we found several ASVs from *acdS*^+^ Proteobacteria and Actinobacteria enriched by water deficit conditioning in the conventionally managed soils. Proteobacteria are generally described to be less abundant after drought stress. Indeed, after the conditioning phase, abundances of species belonging to phyla Acidobacteria, Chloroflexi, Firmicutes, and Latescibacteria were significantly increased under drought, while Proteobacteria decreased (Breitkreuz et al., 2021). This negative trend may be balanced by the plant-growth-promoting properties Proteobacteria often possess, like ACC deaminase. For that reason, Proteobacteria are commonly inoculated on plants to improve drought stress tolerance. In line with our results, the enrichment of the *acdS*^+^ *Variovorax paradoxus* under drought as well as its beneficial effect on plant growth under drought was already shown (Belimov et al., 2009; Teijeiro et al., 2019). Several Actinobacteria were also more abundant after water deficit conditioning. Bouffaud et al. (2018) already described the dominance of *acdS*^+^ Actinobacteria in the rhizosphere, and their consistent enrichment in soil by drought is established (Hartman and Tringe, 2019), as they are mostly known to be drought-tolerant, due to their strong cell wall and drought avoidance by spore formation (Pérez Castro et al., 2019). The genus *Streptomyces* was the most abundant *acdS*^+^ genus and its abundance was enhanced by conditioning with limited water. Members of this Gram-positive genus are characterized as drought tolerant and plant-beneficial (Schrey and Tarkka, 2008; Breitkreuz et al., 2020), which we could also confirm within our study. We particularly identified ASVs corresponding to several *Streptomyces* species enriched by water limitation conditioning, including *S. mutabilis, S. resistomycificus, S. scabiei*, and *S. viridochromogenes*. These *Streptomyces* species have a strong potential to affect the growth of wheat and barley. *S. viridochromogenes* has been characterized as the producer of the herbicide phosphinothricin tripeptide (Schwartz et al., 2004), whereas *S. mutabilis* promotes wheat growth and disease resistance (Toumatia et al., 2016). And finally, the biology of *S. scabiei* is complex and isolate specific; whereas pathogenic *S. scabiei* strains produce thaxtomins and suppress plant defense responses causing growth reduction and scab formation in potatoes (Bignell et al., 2010), other strains of the same species produce staurosporine and control effectively wheat take-all disease (Wen et al., 2012). However, their positive impact on the plant under drought has not yet been described.

### Effect of Plant Identity

While the effect of plant identity on the overall community was comparably low, it had a strong effect on the *acdS*^+^ community, as it was suggested in our third hypothesis. This indicates a strong selection by plant species, likely caused by differential rhizodeposition. Wheat root exudates have been described to differ considerably from those of barley, most notably by the abundance and composition of dominant mugineic acids, and also by differences in the relative quantities of amino acids (Fan et al., 2001). Wheat plants seemed to be less impacted by water deficit conditioning, as reflected by their similar plant heights in the two treatments. The associated microbial communities displayed even higher diversity indices under conventional farming. One explanation may come from the use of winter wheat as a model plant during the first year of the experiment, i.e., the conditioning phase. The close relationship between winter and summer wheat, probably selecting specifically adapted taxa, may have favored the growth of summer wheat plants more than barley in the application phase. This implies that by changing the crop, the positive effect of water deficit history decreased. Similarly, Kaisermann et al. (2017) found that drought legacy effects on plant growth were at their strongest in soils that were conditioned with the same plant species.

### Does Niche Selection Explain the History of Water Deficit?

Differences in the water supply were applied during the conditioning, 25 vs. 60% WHC, and a constant water deficit, 25% WHC, during the application phase. Drought legacy effects on bacterial communities have been reported both after consecutive drought periods (Evans et al., 2014; Bastida et al., 2017) like in our experiment, but also after the drought was terminated (de Vries et al., 2018). A well-supported explanation for changes in microbial composition in response to chronic low soil moisture is niche selection (Evans et al., 2014) leading to an enrichment of taxa that are drought tolerant compared to those that are sensitive to drought. As a result, entire soil microbial communities could develop drought tolerance through time (Bastida et al., 2017), which could lead to increased rates of microbial activity at low soil moistures. When the changes are introduced in the context of plant community and plant-soil feedback, the changes may ultimately sustain the beneficial impact of rhizosphere organisms on plant growth (Canarini et al., 2021). In line with the holobiome concept of the extended plant phenotype, these consequent stages of selection may contribute to overall plant fitness upon drought stress (Liu et al., 2020). Our data support the idea of niche selection by two consecutive drought periods and suggests that resistance of the *acdS*^+^ community is sustained by a highly dynamic community structure. These high dynamics is in line with our observations on the maize rhizosphere *acdS*^+^ community; they were strongly modulated by soil type, but also by soil depth (Gebauer et al., 2021) and the developmental stage of maize (Renoud et al., 2020).

## Conclusions and Perspectives

We demonstrated that water deficit can induce a modification of the community of the specialized plant beneficial prokaryotes that remains detectable during a successive period of low soil moisture in the following year. This result adds to the information on rhizosphere microbial selection by drought and emphasizes that plant growth-promoting bacteria can be especially responsive to abiotic stress. Future work should target how long the difference between the conditioned and unconditioned communities holds up, and whether it vanishes after a period of optimal watering. Members of the *acdS*^+^ community were mainly selected from proteobacterial and actinobacterial species pools, which in the overall community made up the majority of rhizosphere-associated microorganisms. Members of many of the identified taxa can be readily isolated by cultivation, and experiments with synthetic communities would reveal their functional potential. That the responses are distinct in bare soils and rhizospheres, and further modulated by the farming conditions, underlines the context-dependency of the community responses, but hints also that a change in community structure might buffer the negative impacts of water deficit. Future work should thus investigate how these drought-adapted rhizosphere *acdS*^+^ communities represent ACC deaminase activity and support plant performance. This can be used to identify tipping points, thresholds of water deficit severity, or length where even the stress-adapted community cannot sustain plant growth.

## Data Availability Statement

The datasets presented in this study can be found in online repositories. The names of the repository/repositories and accession number(s) can be found below: https://www.ncbi.nlm.nih.gov/, PRJNA783187.

## Author Contributions

CB, TR, and MT conceived and designed the experiment. LG, CB, and M-LB performed the laboratory work. LG, AH-B, and M-LB analyzed the data. M-LB, MT, and CB wrote the manuscript with input from all authors. All authors interpreted the results, contributed to revisions, and approved the submission of the manuscript.

## Funding

This work was done with the support of the Helmholtz Centre for Environmental Research-UFZ. AH-B received funding from the German Centre for Integrative Biodiversity Research (iDiv) Halle-Jena- Leipzig of the German Research Foundation (FZT118, 202548816). CB received funding from the Deutsche Bundesstiftung Umwelt (DBU) in the form of a scholarship (AZ: 20015/391).

## Conflict of Interest

The authors declare that the research was conducted in the absence of any commercial or financial relationships that could be construed as a potential conflict of interest.

## Publisher's Note

All claims expressed in this article are solely those of the authors and do not necessarily represent those of their affiliated organizations, or those of the publisher, the editors and the reviewers. Any product that may be evaluated in this article, or claim that may be made by its manufacturer, is not guaranteed or endorsed by the publisher.

## References

[B1] Altermann M. Rinklebe J. Merbach I. Körschens M. Langer U. Hofmann B. (2005). Chernozem-soil of the year 2005. J. Plant Nutr. Soil Sci. 168, 725–740. 10.1002/jpln.200521814

[B2] Anderson M. J. (2001). A new method for non-parametric multivariate analysis of variance. Austral Ecol. 26, 32–46 10.1111/j.1442-9993.2001.01070.pp.x

[B3] Arshad M. Shaharoona B. Mahmood T. (2008). Inoculation with *Pseudomonas* spp. containing ACC-deaminase partially eliminates the effects of drought stress on growth, yield, and ripening of pea (*Pisum sativum* L.). Pedosphere 18, 611–620. 10.1016/S1002-0160(08)60055-7

[B4] Backer R. Rokem J. S. Ilangumaran G. Lamont J. Praslickova D. Ricci E.. (2018). Plant growth-promoting rhizobacteria: Context, mechanisms of action, and roadmap to commercialization of biostimulants for sustainable agriculture. Front. Plant Sci. 9, 1473. 10.3389/fpls.2018.01473 30405652 PMC6206271

[B5] Balota M. Cristescu S. Payne W. A. te Lintel Hekkert S. Laarhoven L. J. Harren F. J. (2004). Ethylene production of two wheat cultivars exposed to desiccation, heat, and paraquat-induced oxidation. Crop Sci. 44, 812–818. 10.2135/cropsci2004.8120

[B6] Bastida F. Torres I. F. Andrés-Abellán M. Baldrian P. López-Mondéjar R. Větrovský T.. (2017). Differential sensitivity of total and active soil microbial communities to drought and forest management. Glob. Chang. Biol. 23, 4185–4203. 10.1111/gcb.13790 28614633

[B7] Belimov A. A. Dodd I. C. Hontzeas N. Theobald J. C. Safronova V. I. Davies W. J. (2009). Rhizosphere bacteria containing 1-aminocyclopropane-1-carboxylate deaminase increase yield of plants grown in drying soil via both local and systemic hormone signaling. New Phytol. 181, 413–423. 10.1111/j.1469-8137.2008.02657.x 19121036

[B8] Bennett J. A. Klironomos J. (2019). Mechanisms of plant–soil feedback: interactions among biotic and abiotic drivers. New Phytol. 222, 91–96. 10.1111/nph.15603 30451287

[B9] Berendsen R. L. Pieterse C. M. J. Bakker P. A. H. M. (2012). The rhizosphere microbiome and plant health. Trends Plant Sci. 17, 478–486. 10.1016/j.tplants.2012.04.001 22564542

[B10] Berg G. Smalla K. (2009). Plant species and soil type cooperatively shape the structure and function of microbial communities in the rhizosphere. FEMS Microbiol. Ecol. 68, 1–13. 10.1111/j.1574-6941.2009.00654.x 19243436

[B11] Bever J. D. Westover K. M. Antonovics J. (1997). Incorporating the soil community into plant population dynamics: the utility of the feedback approach. J. Ecol. 85, 561–573. 10.2307/2960528

[B12] Bignell D. R. Huguet-Tapia J. C. Joshi M. V. Pettis G. S. Loria R. (2010). What does it take to be a plant pathogen: genomic insights from *Streptomyces* species. Antonie Van Leeuwenhoek 98, 179–194. 10.1007/s10482-010-9429-1 20396949

[B13] Bouffaud M.-L. Renoud S. Dubost A. Moënne-Loccoz Y. Muller D. (2018). 1-Aminocyclopropane-1-carboxylate deaminase producers associated to maize and other Poaceae species. Microbiome 6, 114. 10.1186/s40168-018-0503-7 29925415 PMC6011333

[B14] Breitkreuz C. Buscot F. Tarkka M. Reitz T. (2020). Shifts between and among populations of wheat rhizosphere *Pseudomonas, Streptomyces* and *Phyllobacterium* suggest consistent phosphate mobilization at different wheat growth stages under abiotic stress. Front. Microbiol. 10, 3109. 10.3389/fmicb.2019.03109 32038552 PMC6987145

[B15] Breitkreuz C. Herzig L. Buscot F. Reitz T. Tarkka M. (2021). Interactions between soil properties, agricultural management and cultivar type drive structural and functional adaptations of the wheat rhizosphere microbiome to drought. Environ. Microbiol. 23, 5866–5882. 10.1111/1462-2920.15607 34029439

[B16] Callahan B. J. McMurdie P. J. Rosen M. J. Han A. W. Johnson A. J. A. Holmes S. P. (2016). DADA2: high-resolution sample inference from Illumina amplicon data. Nat. Methods 13, 581–583. 10.1038/nmeth.3869 27214047 PMC4927377

[B17] Canarini A. Schmidt H. Fuchslueger L. Martin V. Herbold C. W. Zezula D.. (2021). Ecological memory of recurrent drought modifies soil processes via changes in soil microbial community. Nat. Commun. 12, 5308. 10.1038/s41467-021-25675-4 34489463 PMC8421443

[B18] Caporaso J. G. Lauber C. L. Walters W. A. Berg-Lyons D. Lozupone C. A. Turnbaugh P. J.. (2011). Global patterns of 16S rRNA diversity at a depth of millions of sequences per sample. Proc. Natl. Acad. Sci. U. S. A. 108, 4516–4522. 10.1073/pnas.1000080107 20534432 PMC3063599

[B19] Danish S. Zafar-ul-Hye M. Mohsin F. Hussain M. (2020). ACC-deaminase producing plant growth promoting rhizobacteria and biochar mitigate adverse effects of drought stress on maize growth. PLoS ONE 15, e0230615. 10.1371/journal.pone.0230615 32251430 PMC7135286

[B20] De Long J. R. Fry E. L. Veen G. F. Kardol P. (2019). Why are plant–soil feedbacks so unpredictable, and what to do about it? Funct. Ecol. 33, 118–128. 10.1111/1365-2435.13232

[B21] de Nijs E. A. Hicks L. C. Leizeaga A. Tietema A. Rousk J. (2019). Soil microbial moisture dependences and responses to drying–rewetting: the legacy of 18 years drought. Glob. Chang. Biol. 25, 1005–1015. 10.1111/gcb.14508 30387912

[B22] de Vries F. T. Griffiths R. I. Bailey M. Craig H. Girlanda M. Gweon H. S.. (2018). Soil bacterial networks are less stable under drought than fungal networks. Nat. Commun. 9, 1–12. 10.1038/s41467-018-05516-7 30072764 PMC6072794

[B23] de Vries F. T. Griffiths R. I. Knight C. G. Nicolitch O. Williams A. (2020). Harnessing rhizosphere microbiomes for drought-resilient crop production. Science 368, 270–274. 10.1126/science.aaz5192 32299947

[B24] European Union (2007). Council regulation (EC) No 834/2007 of 28 June 2007 on organic production and labelling of organic products and repealing regulation (EEC) No 2092/91. Official Journal of the European Union L189/1-23, Luxembourg.

[B25] Evans S. E. Matthew D. Wallenstein Ingrid C. Burke. (2014). Is bacterial moisture niche a good predictor of shifts in community composition under long term drought? Ecology 95, 110–122. 10.1890/13-0500.1 24649651

[B26] Fahad S. Bajwa A. A. Nazir U. Anjum S. A. Farooq A. Zohaib A.. (2017). Crop production under drought and heat stress: plant responses and management options. Front. Plant Sci. 8, 1147. 10.3389/fpls.2017.01147 28706531 PMC5489704

[B27] Fan T. W.-M. Lane A. N. Shenker M. Bartley J. P. Crowley D. Higashi R. M. (2001). Comprehensive chemical profiling of gramineous plant root exudates using high-resolution NMR and MS. Phytochemistry 57, 209–221. 10.1016/S0031-9422(01)00007-3 11382236

[B28] Fish J. Chai B. Wang Q. Sun Y. Brown C. T. Tiedje J.. (2013). FunGene: the functional gene pipeline and repository. Front. Microbiol. 4, 291. 10.3389/fmicb.2013.00291 24101916 PMC3787254

[B29] Gamalero E. Glick B. R. (2012). Ethylene and abiotic stress tolerance in plants, in Environmental Adaptations and Stress Tolerance of Plants in the Era of Climate Change, eds Ahmad P. Prasad M. N. V. (New York, NY: Springer), 395–412. 10.1007/978-1-4614-0815-4_18

[B30] Gargallo-Garriga A. Preece C. Sardans J. Oravec M. Urban O. Peñuelas J. (2018). Root exudate metabolomes change under drought and show limited capacity for recovery. Sci. Rep. 8, 12696. 10.1038/s41598-018-30150-0 30140025 PMC6107494

[B31] Gebauer L. Bouffaud M.-L. Ganther M. Yim B. Vetterlein D. Smalla K.. (2021). Soil texture, sampling depth and root hairs shape the structure of ACC deaminase bacterial community composition in maize rhizosphere. Front. Microbiol. 12:616828. 10.3389/fmicb.2021.616828 33613486 PMC7891401

[B32] Glick B. R. (2005). Modulation of plant ethylene levels by the bacterial enzyme ACC deaminase. FEMS Microbiol. Lett. 251, 1–7. 10.1016/j.femsle.2005.07.030 16099604

[B33] Glick B. R. (2014). Bacteria with ACC deaminase can promote plant growth and help to feed the world. Microbiol. Res. 169, 30–39. 10.1016/j.micres.2013.09.009 24095256

[B34] Gomiero T. Pimentel D. Paoletti M. G. (2011). Environmental impact of different agricultural management practices: conventional vs. organic agriculture. Crit. Rev. Plant Sci. 30, 95–124. 10.1080/07352689.2011.55435529073935

[B35] Gowtham H. G. Singh B. Murali M. Shilpa N. Prasad M. Aiyaz M.. (2020). Induction of drought tolerance in tomato upon the application of ACC deaminase producing plant growth promoting rhizobacterium *Bacillus subtilis* Rhizo SF 48. Microbiol. Res. 234, 126422. 10.1016/j.micres.2020.126422 32058314

[B36] Griffiths B. S. Philippot L. (2013). Insights into the resistance and resilience of the soil microbial community. FEMS Microbiol. Rev. 37, 112–129. 10.1111/j.1574-6976.2012.00343.x 22568555

[B37] Hari V. Rakovec O. Markonis Y. Hanel M. Kumar R. (2020). Increased future occurrences of the exceptional 2018–2019 Central European drought under global warming. Sci. Rep. 10, 12207. 10.1038/s41598-020-68872-9 32764540 PMC7413549

[B38] Harkes P. Suleiman A. K. A. van den Elsen S. J. J. de Haan J. J. Holterman M. Kuramae E. E.. (2019). Conventional and organic soil management as divergent drivers of resident and active fractions of major soil food web constituents. Sci. Rep. 9, 13521. 10.1038/s41598-019-49854-y 31534146 PMC6751164

[B39] Hartman K. Tringe S. G. (2019). Interactions between plants and soil shaping the root microbiome under abiotic stress. Biochem. J. 476, 2705–2724. 10.1042/BCJ20180615 31654057 PMC6792034

[B40] Hartmann M. Frey B. Mayer J. Mäder P. Widmer F. (2015). Distinct soil microbial diversity under long-term organic and conventional farming. ISME J. 9, 1177–1194. 10.1038/ismej.2014.210 25350160 PMC4409162

[B41] Hoagland D. R. Snyder W. C. (1933). Nutrition of strawberry plant under controlled conditions. (a) Effects of deficiencies of boron and certain other elements, (b) susceptibility to injury from sodium salts. Proc. Am. Soc. Horticult. Sci. 30, 288–294.

[B42] Hole D. G. Perkins A. J. Wilson J. D. Alexander I. H. Grice P. V. Evans A. D. (2005). Does organic farming benefit biodiversity? Biol. Conserv. 122, 113–130. 10.1016/j.biocon.2004.07.018

[B43] Jaemsaeng R. Jantasuriyarat C. Thamchaipenet A. (2018). Molecular interaction of 1-aminocyclopropane-1-carboxylate deaminase (ACCD)-producing endophytic *Streptomyces* sp. GMKU towards salt-stress resistance of *Oryza sativa* L. cv. KDML105. Sci. Rep. 8, 1–15. 10.1038/s41598-018-19799-9 29386629 PMC5792428

[B44] Jangid K. Williams M. A. Franzluebbers A. J. Sanderlin J. S. Reeves J. H. Jenkins M. B.. (2008). Relative impacts of land-use, management intensity and fertilization upon soil microbial community structure in agricultural systems. Soil Biol. Biochem. 40, 2843–2853. 10.1016/j.soilbio.2008.07.030

[B45] Kaisermann A. de Vries F. T. Griffiths R. I. Bardgett R. D. (2017). Legacy effects of drought on plant–soil feedbacks and plant–plant interactions. New Phytol. 215, 1413–1424. 10.1111/nph.14661 28621813

[B46] Kundel D. Bodenhausen N. Jørgensen H. B. Truu J. Birkhofer K. Hedlund K.. (2020). Effects of simulated drought on biological soil quality, microbial diversity and yields under long-term conventional and organic agriculture. FEMS Microbiol. Ecol. 96, fiaa205. 10.1093/femsec/fiaa205 33016314 PMC7705324

[B47] Lau J. A. Lennon J. T. (2012). Rapid responses of soil microorganisms improve plant fitness in novel environments. Proc. Nat. Acad. Sci. U. S. A. 109, 14058–14062. 10.1073/pnas.1202319109 22891306 PMC3435152

[B48] Leizeaga A. Hicks L. C. Manoharan L. Hawkes C. V. Rousk J. (2021). Drought legacy affects microbial community trait distributions related to moisture along a savannah grassland precipitation gradient. J. Ecol. 109, 3195–3210. 10.1111/1365-2745.13550

[B49] Liu H. Brettell L. E. Qiu Z. Singh B. K. (2020). Microbiome-mediated stress resistance in plants. Trends Plant Sci. 25:733–743. 10.1016/j.tplants.2020.03.014 32345569

[B50] Livak K. J. Schmittgen T. D. (2001). Analysis of relative gene expression data using real-time quantitative PCR and the 2–ΔΔCT method. Methods 25, 402–408. 10.1006/meth.2001.1262 11846609

[B51] Lori M. Symnaczik S. Mäder P. Deyn G. D. Gattinger A. (2017). Organic farming enhances soil microbial abundance and activity—a meta-analysis and meta-regression. PLoS ONE 12, e0180442. 10.1371/journal.pone.0180442 28700609 PMC5507504

[B52] Love M. I. Huber W. Anders S. (2014). Moderated estimation of fold change and dispersion for RNA-seq data with DESeq2. Genome Biol. 15, 550. 10.1186/s13059-014-0550-8 25516281 PMC4302049

[B53] Lugtenberg B. Kamilova F. (2009). Plant-growth-promoting rhizobacteria. Annu. Rev. Microbiol. 63, 541–556. 10.1146/annurev.micro.62.081307.162918 19575558

[B54] Lupatini M. Korthals G. W. de Hollander M. Janssens T. K. S. Kuramae E. E. (2017). Soil microbiome is more heterogeneous in organic than in conventional farming system. Front. Microbiol. 7, 2064. 10.3389/fmicb.2016.02064 28101080 PMC5209367

[B55] Macias-Benitez S. Garcia-Martinez A. M. Caballero Jimenez P. Gonzalez J. M. Tejada Moral M. Parrado Rubio J. (2020). Rhizospheric organic acids as biostimulants: monitoring feedbacks on soil microorganisms and biochemical properties. Front. Plant Sci. 11, 633. 10.3389/fpls.2020.00633 32547578 PMC7270406

[B56] Marasco R. Rolli E. Ettoumi B. Vigani G. Mapelli F. Borin S.. (2012). A drought resistance-promoting microbiome is selected by root system under desert farming. PLoS ONE 7, e48479. 10.1371/journal.pone.0048479 23119032 PMC3485337

[B57] Mattoo A. K. Suttle J. C. (2017). The Plant Hormone Ethylene. Boca Raton, FL: CRC press. 10.1201/9781351075763

[B58] Mendes L. W. Kuramae E. E. Navarrete A. A. van Veen J. A. Tsai S. M. (2014). Taxonomical and functional microbial community selection in soybean rhizosphere. ISME J. 8, 1577–1587. 10.1038/ismej.2014.17 24553468 PMC4817605

[B59] Morgan P. W. Drew M. C. (1997). Ethylene and plant responses to stress. Physiol. Plant. 100, 620–630. 10.1111/j.1399-3054.1997.tb03068.x

[B60] Munoz-Ucros J. Wilhelm R. C. Buckley D. H. Bauerle T. L. (2022). Drought legacy in rhizosphere bacterial communities alters subsequent plant performance. Plant Soil. 471, 443–461. 10.1007/s11104-021-05227-x

[B61] Murali M. Singh S. B. Gowtham H. G. Shilpa N. Prasad M. Aiyaz M.. (2021). Induction of drought tolerance in *Pennisetum glaucum* by ACC deaminase producing PGPR-*Bacillus amyloliquefaciens* through Antioxidant defense system. Microbiol. Res. 253, 126891. 10.1016/j.micres.2021.126891 34656832

[B62] Naylor D. Coleman-Derr D. (2018). Drought stress and root-associated bacterial communities. Frontiers in Plant Science 8. 10.3389/fpls.2017.02223 29375600 PMC5767233

[B63] Pérez Castro S. Cleland E. E. Wagner R. Sawad R. A. Lipson D. A. (2019). Soil microbial responses to drought and exotic plants shift carbon metabolism. ISME J. 13, 1776–1787. 10.1038/s41396-019-0389-9 30872806 PMC6776022

[B64] Preece C. Peñuelas J. (2016). Rhizodeposition under drought and consequences for soil communities and ecosystem resilience. Plant Soil 409, 1–17. 10.1007/s11104-016-3090-z

[B65] Quast C. Pruesse E. Yilmaz P. Gerken J. Schweer T. Yarza P.. (2013). The SILVA ribosomal RNA gene database project: improved data processing and web-based tools. Nucleic Acids Res. 41, D590–D596. 10.1093/nar/gks1219 23193283 PMC3531112

[B66] Raaijmakers J. M. Paulitz T. C. Steinberg C. Alabouvette C. Moënne-Loccoz Y. (2009). The rhizosphere: a playground and battlefield for soilborne pathogens and beneficial microorganisms. Plant Soil 321, 341–361. 10.1007/s11104-008-9568-6

[B67] Renoud S. Bouffaud M.-L. Dubost A. Prigent-Combaret C. Legendre L. Moënne-Loccoz Y.. (2020). Co-occurrence of rhizobacteria with nitrogen fixation and/or 1-aminocyclopropane-1-carboxylate deamination abilities in the maize rhizosphere. FEMS Microbiol. Ecol. 96, fiaa062. 10.1093/femsec/fiaa062 32275303

[B68] Sanaullah M. Chabbi A. Rumpel C. Kuzyakov Y. (2012). Carbon allocation in grassland communities under drought stress followed by 14C pulse labeling. Soil Biol. Biochem. 55, 132–139. 10.1016/j.soilbio.2012.06.004

[B69] Santos-Medellín C. Edwards J. Liechty Z. Nguyen B. Sundaresan V. (2017). Drought stress results in a compartment-specific restructuring of the rice root-associated microbiomes. MBio 8, e00764–e00717. 10.1128/mBio.00764-17 28720730 PMC5516253

[B70] Schädler M. Buscot F. Klotz S. Reitz T. Durka W. Bumberger J.. (2019). Investigating the consequences of climate change under different land-use regimes: a novel experimental infrastructure. Ecosphere 10, e02635. 10.1002/ecs2.2635

[B71] Schloss P. D. Westcott S. L. Ryabin T. Hall J. R. Hartmann M. Hollister E. B.. (2009). Introducing mothur: open-source, platform-independent, community-supported software for describing and comparing microbial communities. Appl. Environ. Microbiol. 75, 7537–7541. 10.1128/AEM.01541-09 19801464 PMC2786419

[B72] Schrey S. D. Tarkka M. T. (2008). Friends and foes: streptomycetes as modulators of plant disease and symbiosis. Antonie Van Leeuwenhoek 94, 11–19. 10.1007/s10482-008-9241-3 18418729

[B73] Schwartz D. Berger S. Heinzelmann E. Muschko K. Welzel K. Wohlleben W. (2004). Biosynthetic gene cluster of the herbicide phosphinothricin tripeptide from *Streptomyces viridochromogenes* Tu494. Appl. Environ. Microbiol. 70, 7093–7102. 10.1128/AEM.70.12.7093-7102.2004 15574905 PMC535184

[B74] Shakir M. A. Bano A. Arshad M. (2012). Rhizosphere bacteria containing ACC-deaminase conferred drought tolerance in wheat grown under semi-arid climate. Soil Environ. 31, 108–112.

[B75] Song F. Han X. Zhu X. Herbert S. J. (2012). Response to water stress of soil enzymes and root exudates from drought and non-drought tolerant corn hybrids at different growth stages. Can. J. Soil Sci. 92, 501–507. 10.4141/cjss2010-057

[B76] Spinoni J. Vogt J. V. Naumann G. Barbosa P. Dosio A. (2018). Will drought events become more frequent and severe in Europe? Int. J. Climatol. 38, 1718–1736. 10.1002/joc.529124838398

[B77] Tanimoto M. Roberts K. Dolan L. (1995). Ethylene is a positive regulator of root hair development in *Arabidopsis thaliana*. Plant J. 8, 943–948. 10.1046/j.1365-313X.1995.8060943.x 8580964

[B78] Teijeiro R. G. Belimov A. A. Dodd I. C. (2019). Microbial inoculum development for ameliorating crop drought stress: a case study of *Variovorax paradoxus* 5C-2. N. Biotechnol. 56, 103–113. 10.1016/j.nbt.2019.12.006 31899322

[B79] Thakur M. P. Phillips H. R. P. Brose U. De Vries F. T. Lavelle P. Loreau M.. (2020). Towards an integrative understanding of soil biodiversity. Biol. Rev. 95, 350–364. 10.1111/brv.12567 31729831 PMC7078968

[B80] Toumatia O. Compant S. Yekkour A. Goudjal Y. Sabaou N. Mathieu F.. (2016). Biocontrol and plant growth promoting properties of *Streptomyces mutabilis* strain IA1 isolated from a Saharan soil on wheat seedlings and visualization of its niches of colonization. South Afr. J. Bot. 105, 234–239. 10.1016/j.sajb.2016.03.020

[B81] Vacheron J. Desbrosses G. Bouffaud M.-L. Touraine B. Moënne-Loccoz Y. Muller D.. (2013). Plant growth-promoting rhizobacteria and root system functioning. Front. Plant Sci. 4, 356. 10.3389/fpls.2013.00356 24062756 PMC3775148

[B82] Veach A. M. Zeglin L. H. (2020). Historical drought affects microbial population dynamics and activity during soil drying and re-wet. Microb. Ecol. 79, 662–674. 10.1007/s00248-019-01432-5 31482287

[B83] Vurukonda S. S. K. P. Vardharajula S. Shrivastava M. Sk Z. A. (2016). Enhancement of drought stress tolerance in crops by plant growth promoting rhizobacteria. Microbiol. Res. 184, 13–24. 10.1016/j.micres.2015.12.003 26856449

[B84] Weißbecker C. Schnabel B. Heintz-Buschart A. (2020). Dadasnake, a Snakemake implementation of DADA2 to process amplicon sequencing data for microbial ecology. Gigascience 9, giaa135. 10.1093/gigascience/giaa135 33252655 PMC7702218

[B85] Wen C. Zheng D. Shen S. Chen J. Liu W. Liu T. (2012). *Streptomyces scabiei* subsp. xuchangensis, a novel Streptomycete isolate for staurosporine production and a wheat take-all control agent. Int. J. Microbiol. Res. 4, 282–289. 10.9735/0975-5276.4.7.282-289

[B86] Yuan J. Zhao J. Wen T. Zhao M. Li R. Goossens P.. (2018). Root exudates drive the soil-borne legacy of aboveground pathogen infection. Microbiome 6, 156. 10.1186/s40168-018-0537-x 30208962 PMC6136170

[B87] Zhalnina K. Louie K. B. Hao Z. Mansoori N. da Rocha U. N. Shi S.. (2018). Dynamic root exudate chemistry and microbial substrate preferences drive patterns in rhizosphere microbial community assembly. Nat. Microbiol. 3, 470–480. 10.1038/s41564-018-0129-3 29556109

[B88] Zilber-Rosenberg I. Rosenberg E. (2008). Role of microorganisms in the evolution of animals and plants: the hologenome theory of evolution. FEMS Microbiol. Rev. 32, 723–735. 10.1111/j.1574-6976.2008.00123.x 18549407

