# Supplementary material for: Water Deficit History Selects Plant Beneficial Soil Bacteria Differently Under Conventional and Organic Farming
Source: Front Microbiol. 2022 Jun 13;13:824437. doi: 10.3389/fmicb.2022.824437 (PMC9234553; doi:10.3389/fmicb.2022.824437)
Supplement: Supplementary file 1 [file Data_Sheet_1.docx]

Supplementary Material

**Supplementary Table 1.** Results of three-way ANOVAs for the dominant taxa within the 16S rRNA and *acdS* data sets. The relative abundance of the genera was considered for all treatments, to determine the effect of plant (wheat, barley and bulk soil), farming system (organic and conventional farming) and watering condition during the conditioning phase (CD and DD), and *P*-values are indicated.

|  |  | **Farming (F)** | | | **Plant (P)** | | **Conditioning (C)** | | **F x P** | | **F x C** | | **P x C** | | **F x P x C** | |
| --- | --- | --- | --- | --- | --- | --- | --- | --- | --- | --- | --- | --- | --- | --- | --- | --- |
| acdS | |  | | |  | |  | |  | |  | |  | |  | |
|  | u. Bacteria | | 0.361 | | **< 0.001** | | 0.63 | | **0.006** | | 0.379 | | **0.018** | | 0.751 | |
|  | Variovorax | | **0.005** | | **< 0.001** | | 0.133 | | 0.394 | | 0.73 | | 0.09 | | 0.628 | |
|  | Acidovorax | | **0.01** | | **< 0.001** | | **0.021** | | 0.788 | | 0.582 | | 0.136 | | 0.093 | |
|  | Methylibium | | **0.017** | | **< 0.001** | | 0.47 | | 0.82 | | 0.216 | | 0.242 | | 0.321 | |
|  | Burkholderia | | 0.872 | | 0.864 | | 0.118 | | 0.63 | | 0.299 | | 0.208 | | 0.63 | |
|  | Ralstonia | | **0.002** | | **< 0.001** | | **0.024** | | 0.766 | | 0.056 | | 0.125 | | 0.763 | |
|  | Paraburkholderia | | **< 0.001** | | **< 0.001** | | 0.4 | | **0.013** | | 0.564 | | 0.643 | | 0.717 | |
|  | u. Actinobacteria | | **0.009** | | **0.002** | | 0.821 | | 0.431 | | 0.987 | | 0.35 | | 0.555 | |
|  | Actinoplanes | | 0.736 | | **< 0.001** | | **0.004** | | **0.018** | | **0.002** | | **0.024** | | 0.051 | |
|  | Modestobacter | | **< 0.001** | | 0.07 | | 0.519 | | 0.663 | | 0.746 | | **0.038** | | 0.214 | |
|  | Saccharothrix | | **0.031** | | **< 0.001** | | **0.005** | | 0.28 | | **0.027** | | **0.011** | | 0.201 | |
|  | Amycolatopsis | | 0.436 | | 0.151 | | **0.003** | | 0.565 | | **0.03** | | **< 0.001** | | 0.196 | |
|  | Marmoricola | | **< 0.001** | | **< 0.001** | | **< 0.001** | | **< 0.001** | | **< 0.001** | | **0.002** | | **< 0.001** | |
|  | u. Nocardioidaceae | | **< 0.001** | | **< 0.001** | | **< 0.001** | | **< 0.001** | | **< 0.001** | | **< 0.001** | | **< 0.001** | |
|  | Phycicoccus | | **0.009** | | **0.02** | | **0.002** | | **< 0.001** | | 0.493 | | **< 0.001** | | 0.281 | |
|  | Tetrasphaera | | 0.063 | | 0.573 | | **< 0.001** | | 0.563 | | 0.795 | | **0.009** | | 0.14 | |
|  | Microbacterium | | **< 0.001** | | **< 0.001** | | **< 0.001** | | 0.172 | | 0.434 | | 0.315 | | 0.612 | |
|  | Plantibacter | | **0.009** | | **< 0.001** | | **< 0.001** | | 0.547 | | 0.801 | | **< 0.001** | | 0.331 | |
|  | u. Micrococcales | | 0.066 | | 0.201 | | **< 0.001** | | 0.709 | | 0.523 | | 0.064 | | 0.121 | |
|  | Streptomyces | | 0.269 | | **0.007** | | 0.307 | | 0.196 | | 0.091 | | 0.706 | | 0.616 | |
|  |  | |  | |  | |  | |  | |  | |  | |  | |
| 16S rRNA | | |  |  | |  | |  | |  | |  | |  | |  |
|  | u. Nitrososphaeraceae | | **< 0.001** | | 0.064 | | **0.014** | | 0.333 | | 0.395 | | 0.211 | | 0.748 | |
|  | C. Udeobacter | | 0.673 | | **< 0.001** | | **0.047** | | 0.138 | | 0.18 | | **0.017** | | 0.818 | |
|  | u. Chitinophagaceae | | 0.98 | | **0.002** | | 0.427 | | 0.83 | | 0.196 | | 0.724 | | 0.708 | |
|  | u. Gemmatimonadaceae | | **0.004** | | **< 0.001** | | 0.663 | | **< 0.001** | | 0.331 | | **0.017** | | 0.785 | |
|  | Gemmatimonas | | 0.438 | | **< 0.001** | | 0.111 | | 0.1 | | 0.241 | | 0.949 | | 0.935 | |
|  | Acidobacteria SG6 | | 0.475 | | **< 0.001** | | 0.11 | | 0.952 | | 0.96 | | 0.649 | | 0.497 | |
|  | RB41 | | **0.002** | | **< 0.001** | | **0.007** | | **< 0.001** | | 0.166 | | **0.011** | | 0.129 | |
|  | JG 30 KF CM45 | | 0.411 | | **< 0.001** | | **< 0.001** | | 0.189 | | 0.501 | | **0.009** | | **0.018** | |
|  | KD4 96 | | **< 0.001** | | **< 0.001** | | **< 0.001** | | 0.193 | | 0.856 | | **< 0.001** | | 0.207 | |
|  | SC I 84 | | **< 0.001** | | **< 0.001** | | 0.744 | | **< 0.001** | | 0.507 | | 0.42 | | 0.885 | |
|  | Dyella | | 0.102 | | **< 0.001** | | **< 0.001** | | 0.154 | | 0.64 | | **< 0.001** | | 0.753 | |
|  | Luteimonas | | 0.188 | | **< 0.001** | | 0.783 | | 0.576 | | **< 0.001** | | 0.966 | | **0.038** | |
|  | Rhodanobacter | | **0.004** | | **< 0.001** | | 0.112 | | 0.424 | | 0.384 | | 0.347 | | 0.679 | |
|  | Sphingomonas | | **0.008** | | **< 0.001** | | **< 0.001** | | 0.08 | | 0.708 | | **0.003** | | 0.764 | |
|  | Gaiella | | **0.039** | | **< 0.001** | | 0.72 | | 0.5 | | 0.448 | | **0.004** | | 0.59 | |
|  | u. Gaiellales | | 0.061 | | **0.004** | | 0.356 | | 0.153 | | 0.663 | | **0.008** | | 0.055 | |
|  | u. Solirubrobacterales | | **0.003** | | 0.092 | | **0.012** | | 0.267 | | 0.807 | | **0.02** | | 0.068 | |
|  | Glycomyces | | **0.023** | | **< 0.001** | | 0.225 | | 0.148 | | 0.668 | | 0.685 | | 0.945 | |
|  | Streptomyces | | **< 0.001** | | **< 0.001** | | **0.039** | | **0.014** | | 0.373 | | 0.41 | | 0.591 | |
|  | Nocardioides | | 0.303 | | **< 0.001** | | **0.004** | | 0.554 | | 0.522 | | 0.335 | | 0.965 | |

**Supplementary Table 2.** Results of differential ASV abundance after drought conditioning performed on the *acdS* data set of the rhizosphere samples. The differential abundance was tested using DESeq2 (Love et al., 2014) on the ASVs representing at least 0.05% of the unrarefied reads. Log2 fold changes and p-values are indicated for each of the significant ASVs (P<0.05), as well as their taxonomic affiliation. CD: no water deficit conditioning, DD: water deficit conditioning, OF: organic farming, CF: conventional farming.


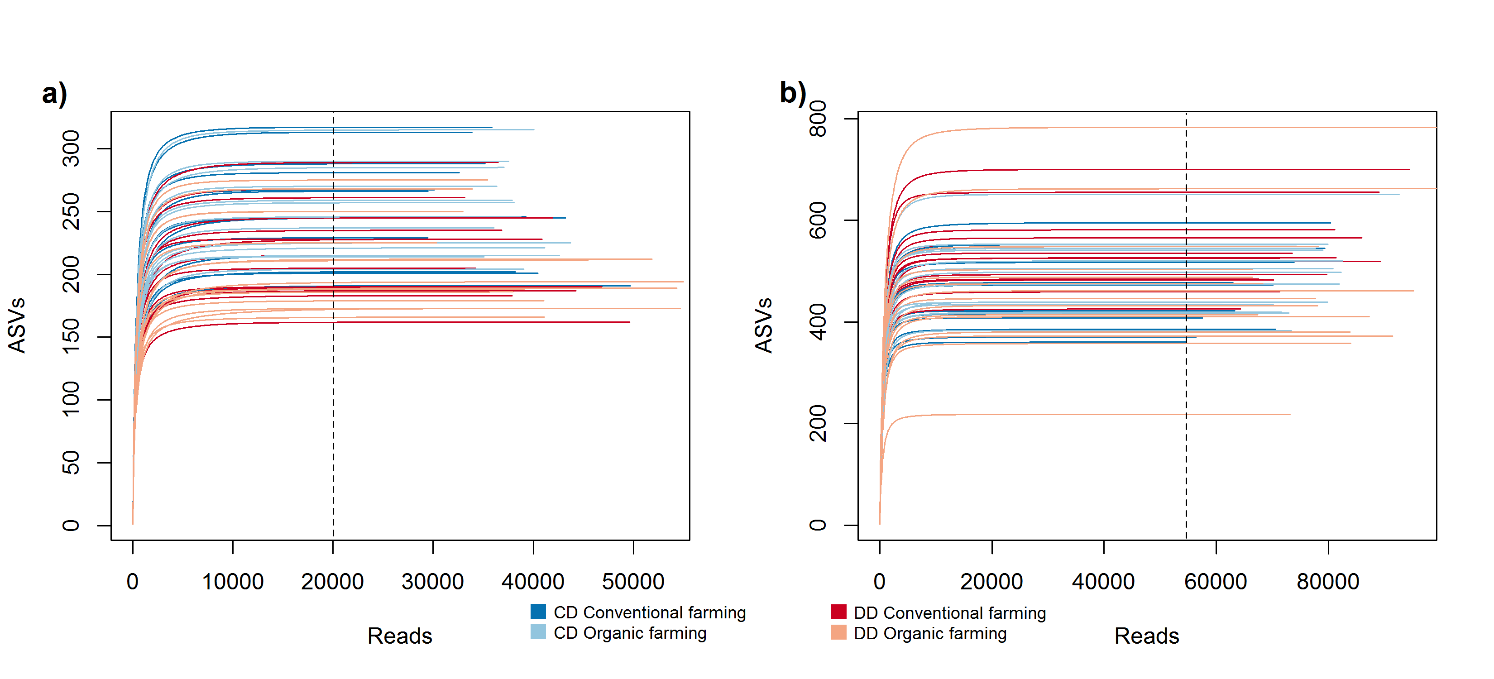


**Supplementary Figure 1.** Rarefaction curves showing the number of ASVs related to the numbers of reads obtained for each sample. a) 16S rRNA gene*,* b) *acdS*. The dotted lines correspond to the minimum number of reads across all treatments, which were used to rarefy the read numbers. CD: no water deficit conditioning, DD: water deficit conditioning.


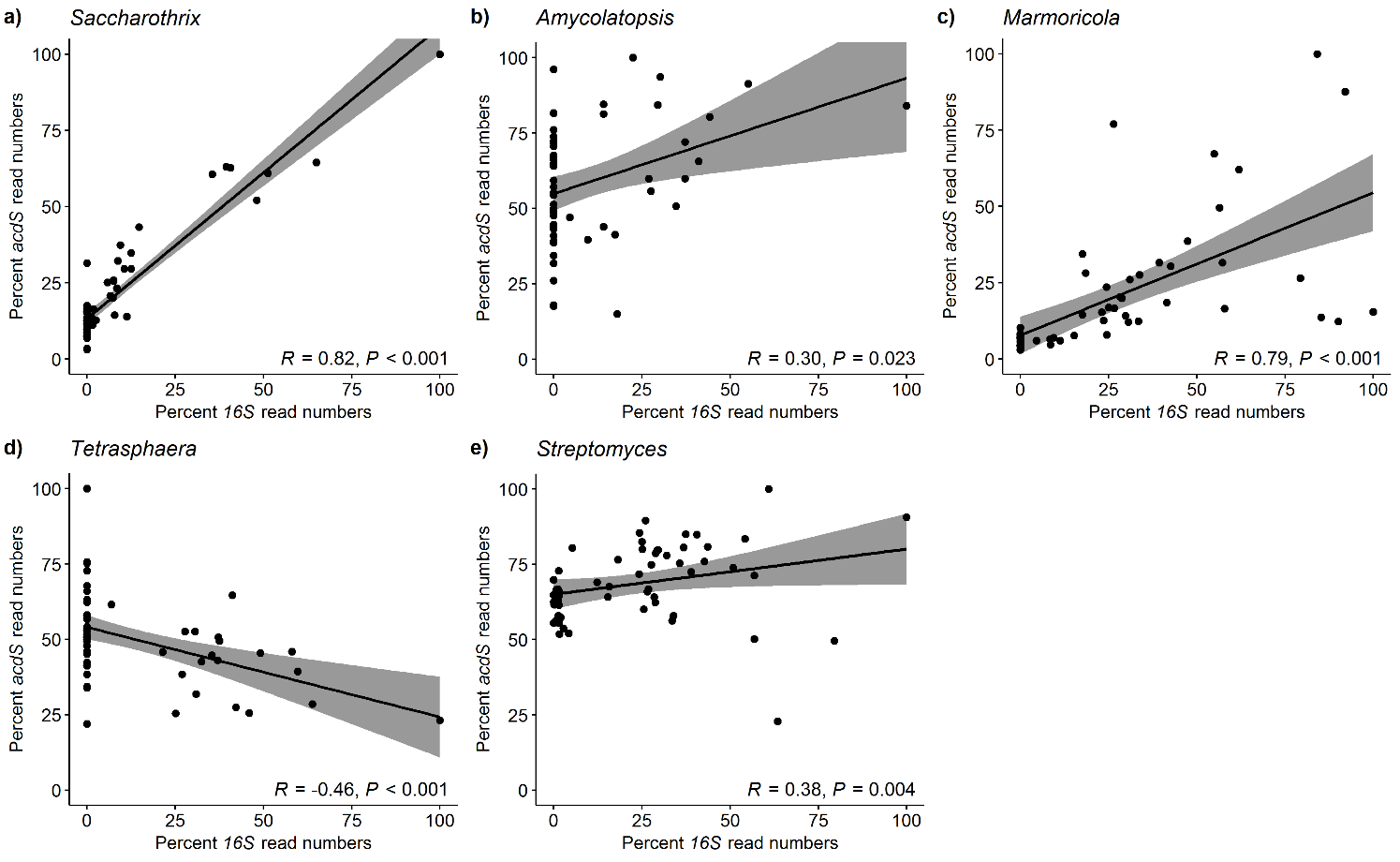


**Supplementary Figure 2.** Correlation between relative abundances of *acdS* and 16S rRNA gene sequences across treatments. Values for *P* and rho (R) of spearman correlation are given for sequences affiliated to a) *Saccharothrix*, b) *Amycolatopsis*, c) *Marmoricola*, d) *Tetrasphaera* and e) *Streptomyces*. The grey shaded area represents the 95% confidence level.


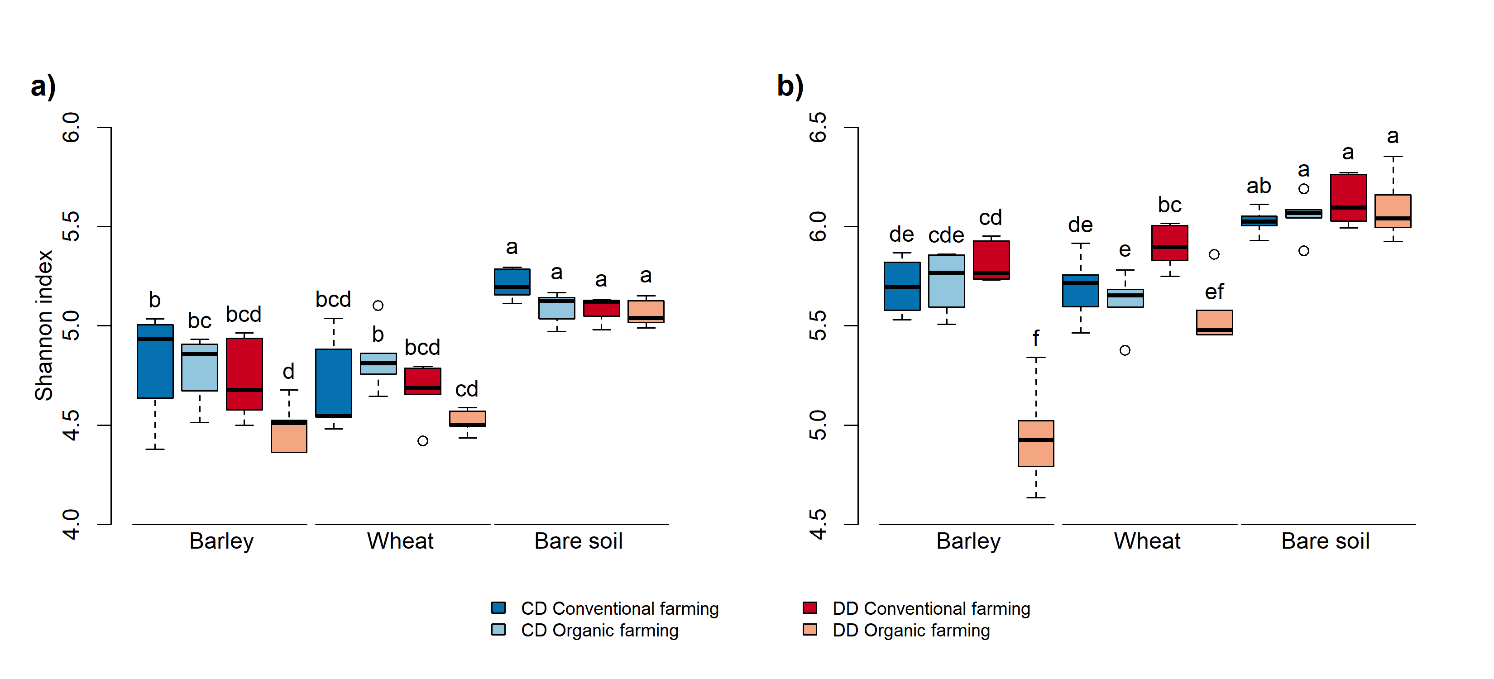


**Supplementary Figure 3.** Boxplots of the Shannon indices obtained for each treatment. a) 16S rRNA genes, b) *acdS*. Letters correspond to the statistical differences between treatments according to Kruskal-Wallis test followed by Fisher´s LSD *post hoc* test with Benjamini-Hochberg *P*-value adjustment. CD: no water deficit conditioning, DD: water deficit conditioning.


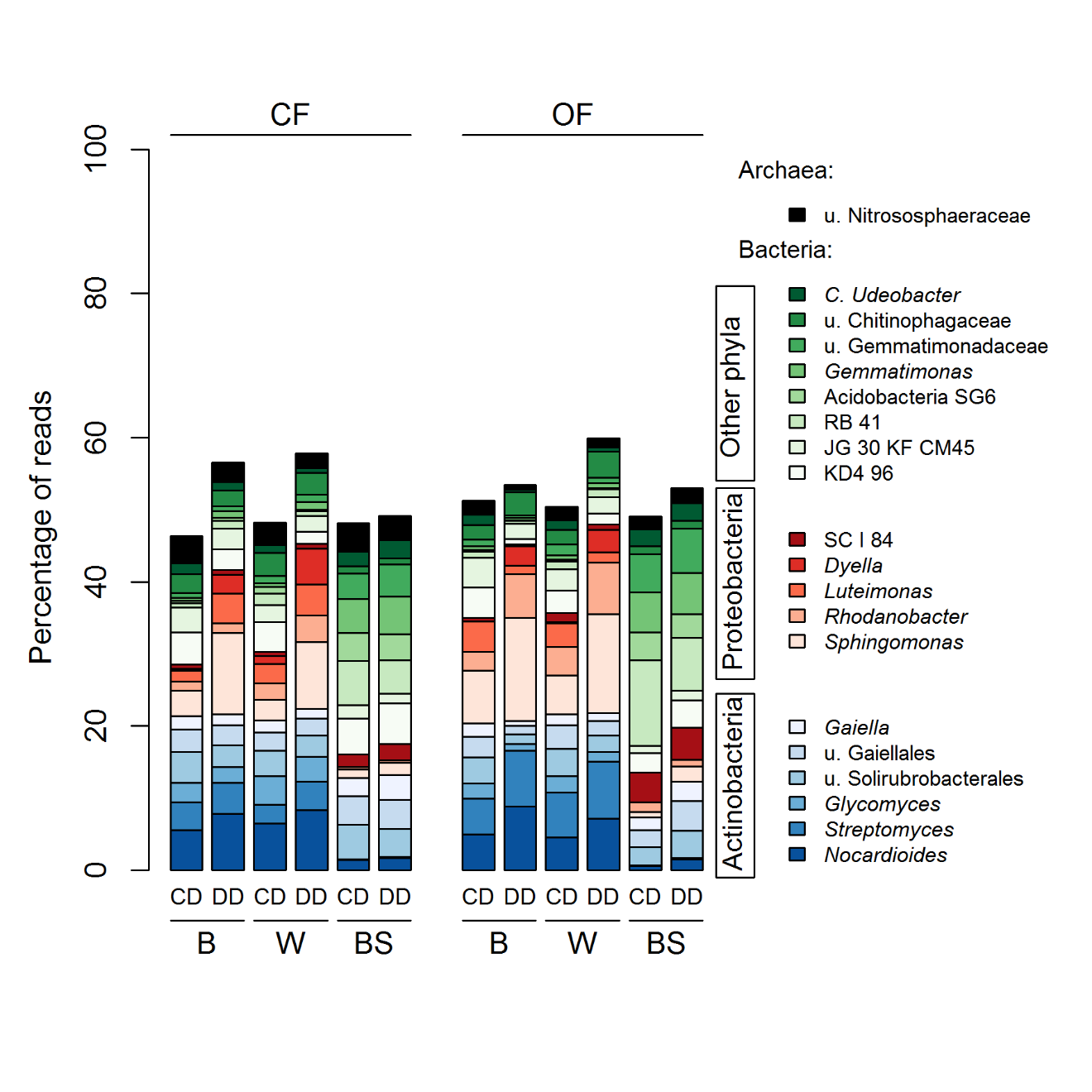


**Supplementary Figure 4.** Distribution of the 20 most abundant genera across all treatments for the 16S rRNA gene dataset. CF: conventional farming, OF: organic farming, B: barley, W: wheat, BS: bare soil, CD: no water deficit conditioning, DD: water deficit conditioning, u.: unclassified, C.: Candidatus.
